# Simulating the progression of brain structural alterations in Parkinson’s disease

**DOI:** 10.1038/s41531-022-00349-0

**Published:** 2022-06-28

**Authors:** Chang-hyun Park, Na-Young Shin, Sang-Won Yoo, Haeseok Seo, Uicheul Yoon, Ji-Yeon Yoo, Kookjin Ahn, Joong-Seok Kim

**Affiliations:** 1 Department of Radiology, College of Medicine, Catholic University of Korea, Seoul, Korea; 2 Center for Neuroprosthetics and Brain Mind Institute, Swiss Federal Institute of Technology (EPFL), Geneva, Switzerland; 3 Department of Neurology, College of Medicine, Catholic University of Korea, Seoul, Korea; 4 Department of Biomedical Engineering, College of Bio and Medical Sciences, Daegu Catholic University, Gyeongsan, Gyeongbuk Korea

**Keywords:** Parkinson's disease, Parkinson's disease

## Abstract

Considering brain structural alterations as neurodegenerative consequences of Parkinson's disease (PD), we sought to infer the progression of PD via the ordering of brain structural alterations from cross-sectional MRI observations. Having measured cortical thinning in gray matter (GM) regions and disintegrity in white matter (WM) regions as MRI markers of structural alterations for 130 patients with PD (69 ± 10 years, 72 men), stochastic simulation based on the probabilistic relationship between the brain regions was conducted to infer the ordering of structural alterations across all brain regions and the staging of structural alterations according to changes in clinical status. The ordering of structural alterations represented WM disintegrity tending to occur earlier than cortical thinning. The staging of structural alterations indicated structural alterations happening mostly before major disease complications such as postural instability and dementia. Later disease states predicted by the sequence of structural alterations were significantly related to more severe clinical symptoms. The relevance of the ordering of brain structural alterations to the severity of clinical symptoms suggests the clinical feasibility of predicting PD progression states.

## Introduction

The characteristic motor symptoms of Parkinson's disease (PD) result from dopaminergic cell loss in the substantia nigra, which involves the accumulation of α-synuclein into Lewy bodies. As the pathological hallmark of the disease, Lewy bodies are often found within the brainstem^1^ and then they are mainly involved in the sequential ascending course of pathology in the brain. The presence of Lewy bodies can occur even before the onset of motor symptoms^2^, and the sequential spreading of Lewy body pathology in the brain tends to be related to the worsening of motor and non-motor symptoms. Despite the crucial role of Lewy body pathology underlying the development of various symptoms of PD, it is yet challenging to detect it in the living brain.

As an alternative to pathological markers, widely available imaging such as MRI provides phenotypic observations on structural alterations in the pathological brain. A wealth of literature suggests brain structural alterations, specifically marked as cortical thinning and white matter (WM) disintegrity, as neurodegenerative consequences of PD (see reviews in refs. ^3–5^). As MRI markers of brain structural alterations related to a loss of brain tissue or its connections, cortical thinning indicates a reduction in the width of cortical gray matter (GM), as usually measured from structural MRI, and WM disintegrity represents a disruption to WM diffusion properties, as usually measured from diffusion-weighted MRI. Multiple lines of evidence support brain structural alterations accompanied by motor and non-motor symptoms, indicating the association between the extent of brain structural alterations and the severity of symptoms, notably progressive cognitive impairment to mild cognitive impairment (MCI) and dementia^6–9^, in patients with PD. However, it has yet to be fully established how such MRI markers could be used in terms of their ordering to track the progression of PD.

Event-based disease progression modeling has been suggested as a way of describing the progression of neurodegenerative diseases as a monotonic progression of events and learning the ordering of the events^10–12^. Although longitudinal follow-up of patients with PD would be crucial in describing PD progression based on a faithful characterization of baseline information, it is often limited by financial or logistical demands that could lead to loss to follow-up^13^. Considering the challenge of longitudinal follow-up of patients with PD, the practical utility of event-based disease progression modeling is in that it can provide a view of longitudinal disease progression from cross-sectional observations across individual patients. Here we employed the notion of event-based disease progression modeling to examine PD progression by including brain structural alterations as events that represented observable changes in the state of a patient. That is, the ordering of brain structural alterations was not observed through longitudinal follow-up but inferred from cross-sectional observations in this study, so that temporal precedence reflected by the ordering would be hypothetical.

Cross-sectional observations of brain regions at different status of structural alterations across individual patients may be formulated as probabilistic relationships of structural alterations between the brain regions. For example, when the presence (+) or absence (−) of a structural alteration in two brain regions, *X* and *Y*, is considered, the probabilistic relationship between the two brain regions can be expressed by conditional probabilities, *P*(*X*+| *Y*−) and *P*(*Y*+| *X*−), which would be specifically informative about the ordering of structural alterations (Supplementary Fig. 1)^14^.

**Fig. 1:**
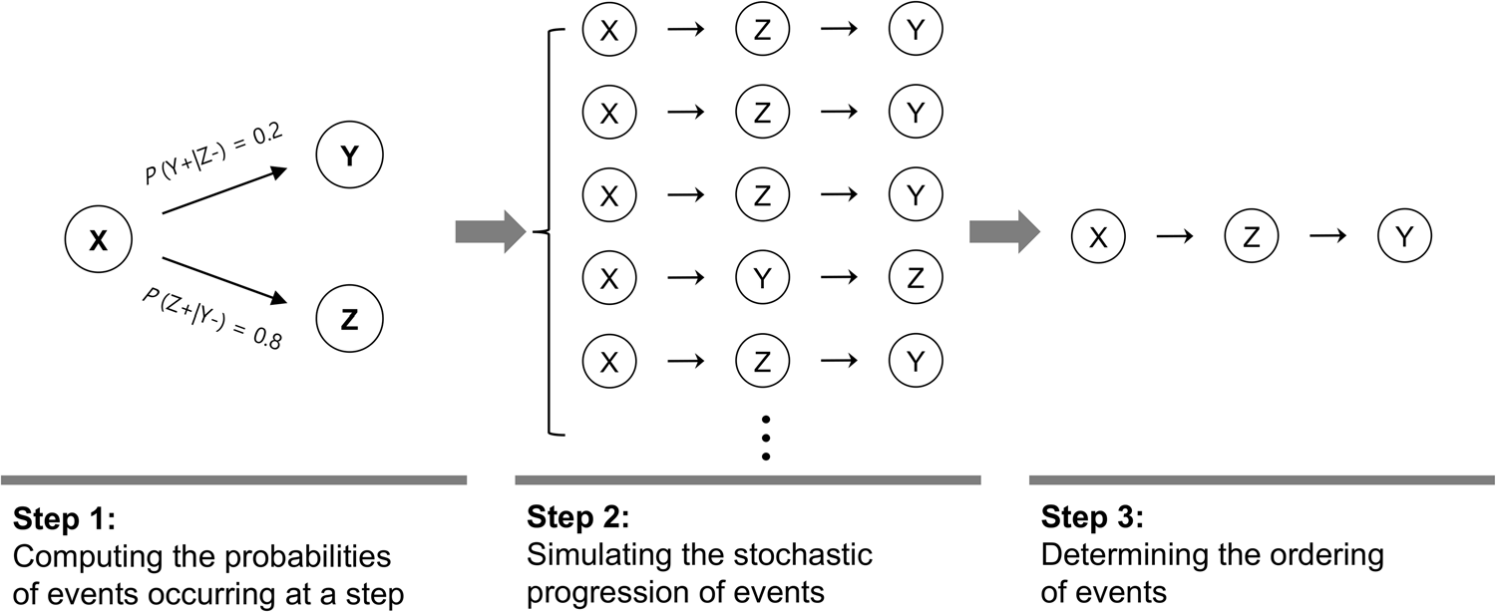
Three steps for inferring the ordering of structural alterations. The example shows that, when the sequential spreading of structural alterations from a brain region, *X*, to two brain regions, *Y* and *Z*, is considered, the higher likelihood of a structural alteration occurring earlier in *Z* than in *Y* could lead to a more frequent occurrence of a structural alteration in *Z* before that in *Y* in a simulation, thus enabling to infer the progression of structural alterations from *X* to *Z* to *Y*.

Based on the conditional probability that a structural alteration in one brain region preceded those in other brain regions, we simulated the progression of structural alterations. Following the example above, if the occurrence of structural alterations in the two brain regions, *X* and *Y*, was not seen yet, which brain region between the two brain regions would show a structural alteration at a specific step could be inferred by (i) computing conditional probabilities, *P*(*X*+| *Y*−) and *P*(*Y*+| *X*−), reflecting the individual brain regions’ probabilities of the events occurring at the step, (ii) simulating the stochastic progression of the events via random sampling according to the probabilities, and (iii) determining the ordering of the events based on the frequency of occurrence at the step in the simulation (Fig. 1).

We hypothesized that the stochastic simulation of the progression of structural alterations would enable us to infer the sequence of structural alterations possibly following ascending dopaminergic projections. Furthermore, we supposed that the sequence of structural alterations inferred at the patient cohort level could be used to make predictions of individual patients’ disease states, thus allowing us to assess the association between the progression of structural alterations and the worsening of clinical symptoms at the individual patient level.

## Results

### Patient characteristics

Table 1 summarizes the clinical and neuropsychological characteristics of patients included in this study. Of the 130 patients with PD (69 ± 10 years, 72 men), 68 patients were assessed as Hoehn and Yahr Stage (HYS) < 2 (unilateral symptoms/signs), 56 patients as HYS ≥ 2 and <3 (bilateral symptoms/signs), and 6 patients as HYS ≥ 3 (postural instability) according to the modified Hoehn and Yahr Staging Scale^15^. On the other hand, 54 patients were diagnosed as PD with normal cognition (PD-NC), 70 patients as PD with MCI (PD-MCI), and 6 patients as PD with dementia (PD-D) according to the incidence and severity of cognitive impairment. Thus, most of PD patients were considered as being in early PD in that they were not yet confronted by PD milestones as major determinants of experience of the disease^16^, such as postural instability and dementia.

**Table 1:** Clinical and neuropsychological characteristics of patients with Parkinson's disease (PD) in different cognitive status.

		PD-N (*n* = 54)	PD-MCI (*n* = 70)	PD-D (*n* = 6)	*P* value
Age (year)		67 ± 10	70 ± 9	75 ± 7	0.08
Sex (men:women)		26:28	41:29	5:1	0.20
Education (year)		11 ± 5	11 ± 5	11 ± 4	0.91
Age at onset (year)		66 ± 9	68 ± 9	73 ± 8	0.15
Disease duration (year)		1.3 ± 1.6	1.4 ± 1.4	4.0 ± 2.8	0.04^*,b,c^
Levodopa equivalent daily dose (mg)		71 ± 181	65 ± 185	399 ± 432	0.03^*,b,c^
HYSS		1.4 ± 0.7	1.7 ± 0.7	1.9 ± 0.7	0.09
UPDRS		21.2 ± 15.1	25.1 ± 14.6	49.2 ± 22.3	<0.01^*,b,c^
MMSE		28.0 ± 2.8	26.2 ± 2.9	18.5 ± 4.1	<0.01^*,a,b,c^
GDS		3.9 ± 3.9	5.2 ± 4.4	9.6 ± 4.5	0.02^*,b^
Neuropsychological evaluation	Attention	0.2 ± 0.7	−0.4 ± 0.9	−0.8 ± 0.6	<0.01^*,a,b^
	Memory	0.1 ± 0.6	−0.8 ± 0.7	−2.0 ± 0.7	<0.01^*,a,b,c^
	Language	0.4 ± 1.1	−0.8 ± 2.0	−5.4 ± 4.2	<0.01^*,a,b,c^
	Visuospatial function	0.1 ± 0.7	−1.0 ± 2.0	−4.7 ± 3.4	<0.01^*,a,b,c^
	Executive function	0.2 ± 0.8	−0.6 ± 0.7	−1.8 ± 0.9	<0.01^*,a,b,c^
	Aggregate	0.2 ± 0.4	−0.7 ± 0.8	−2.8 ± 1.4	<0.01^*,a,b,c^

*PD-N* PD with normal cognition, *PD-MCI* PD with mild cognitive impairment, *PD-D* PD with dementia, *HYSS* modified Hoehn and Yahr Staging Scale, *UPDRS* Unified Parkinson’s Disease Rating Scale, *MMSE* Mini-Mental State Examination, *GDS* Geriatric Depression Scale.

Notes: ^*^Significant difference between PD-N, PD-MCI, and PD-D in the Kruskal–Wallis test; ^a^significant difference between PD-N and PD-MCI; ^b^significant difference between PD-N and PD-D; and ^c^significant difference between PD-MCI and PD-D in the Wilcoxon rank-sum test as being post hoc.

When the comparison of clinical and neuropsychological characteristics was made between the patients in different cognitive status by adopting the Kruskal–Wallis test followed by the Wilcoxon rank-sum test as being post hoc, with a significance level of a *P* value ≤ 0.05 after correction for multiple comparisons using a false discovery rate method, the global cognition score was significantly different in terms of both the Seoul Neuropsychological Screening Battery^17^ aggregate *z*-score and Mini-Mental State Examination score, such that cognitive impairment was most severe in PD-D, followed by PD-MCI. Specifically, differences in the Seoul Neuropsychological Screening Battery domain *z*-score between the patients in different cognitive status were observed across five cognitive domains, including attention, memory, language, visuospatial function, and executive function. In addition, disease duration was longer, the daily dose of medications was higher, clinical symptoms in terms of the Unified Parkinson’s Disease Rating Scale (UPDRS)^18^ score were more severe in PD-D than in PD-N and PD-MCI, and depression in terms of the Geriatric Depression Scale score was more severe in PD-D than in PD-N.

### Ordering between cortical thinning and WM disintegrity

**Fig. 2:**
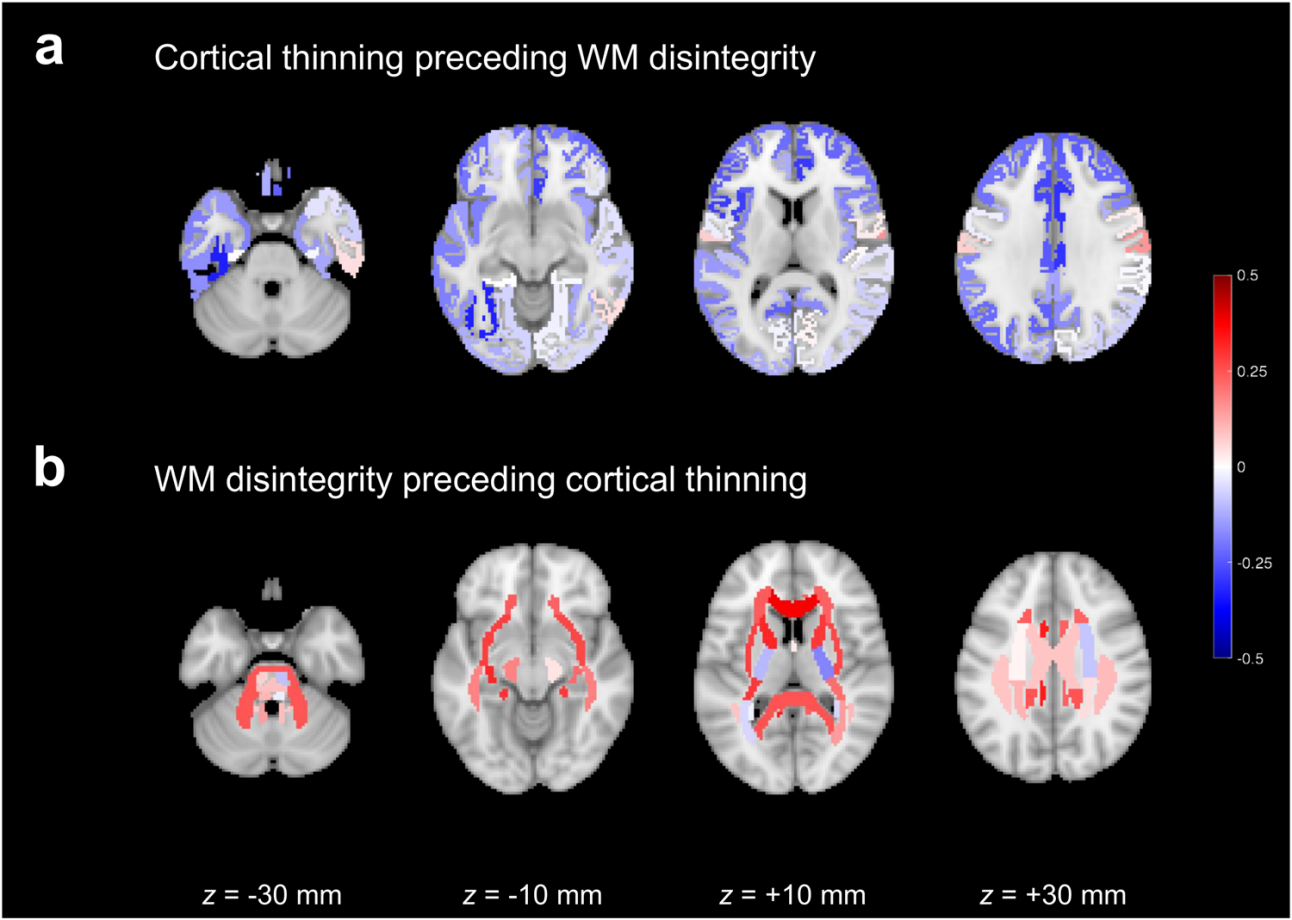
Ordering of structural alterations between cortical gray matter (GM) regions and white matter (WM) regions. **a** The likelihood of cortical thinning preceding WM disintegrity, represented by *P*(GM+| WM−) − *P*(WM+| GM−), in cortical GM regions. **b** The likelihood of WM disintegrity preceding cortical thinning, expressed by *P*(WM+| GM−) − *P*(GM+| WM−), in WM regions. Darker red indicates the earlier occurrence of structural alterations, whereas darker blue represents the later occurrence of structural alterations.

Comparisons of conditional probabilities for regional pair combinations between 62 cortical GM regions and 48 WM regions were significant towards the precedence of WM disintegrity over cortical thinning for 24% (702/2976) of all regional pair combinations and towards the precedence of cortical thinning over WM disintegrity for 5% (142/2976) of all regional pair combinations (Supplementary Fig. 2). Figure 2 and Supplementary Table 1 exhibit the ordering of structural alterations between cortical GM regions and WM regions, in terms of the difference between *P*(GM+| WM−) and *P*(WM+| GM−), where *P*(GM+| WM−) represented the probability of a specific cortical GM region’s structural alteration occurring earlier than WM regions’ structural alterations on average, and *P*(WM+| GM−) expressed the probability of a specific WM region’s structural alteration occurring earlier than cortical GM regions’ structural alterations on average. As depicted in the distribution of probability differences in Supplementary Fig. 3, positive values of *P*(WM+| GM−) − *P*(GM+| WM−) in most of WM regions (40 of 48 WM regions) as well as negative values of *P*(GM+| WM−) − *P*(WM+| GM−) in most of cortical GM regions (55 of 62 cortical GM regions) indicate that WM disintegrity generally preceded cortical thinning.

### Ordering of structural alterations across all brain regions

**Fig. 3:**
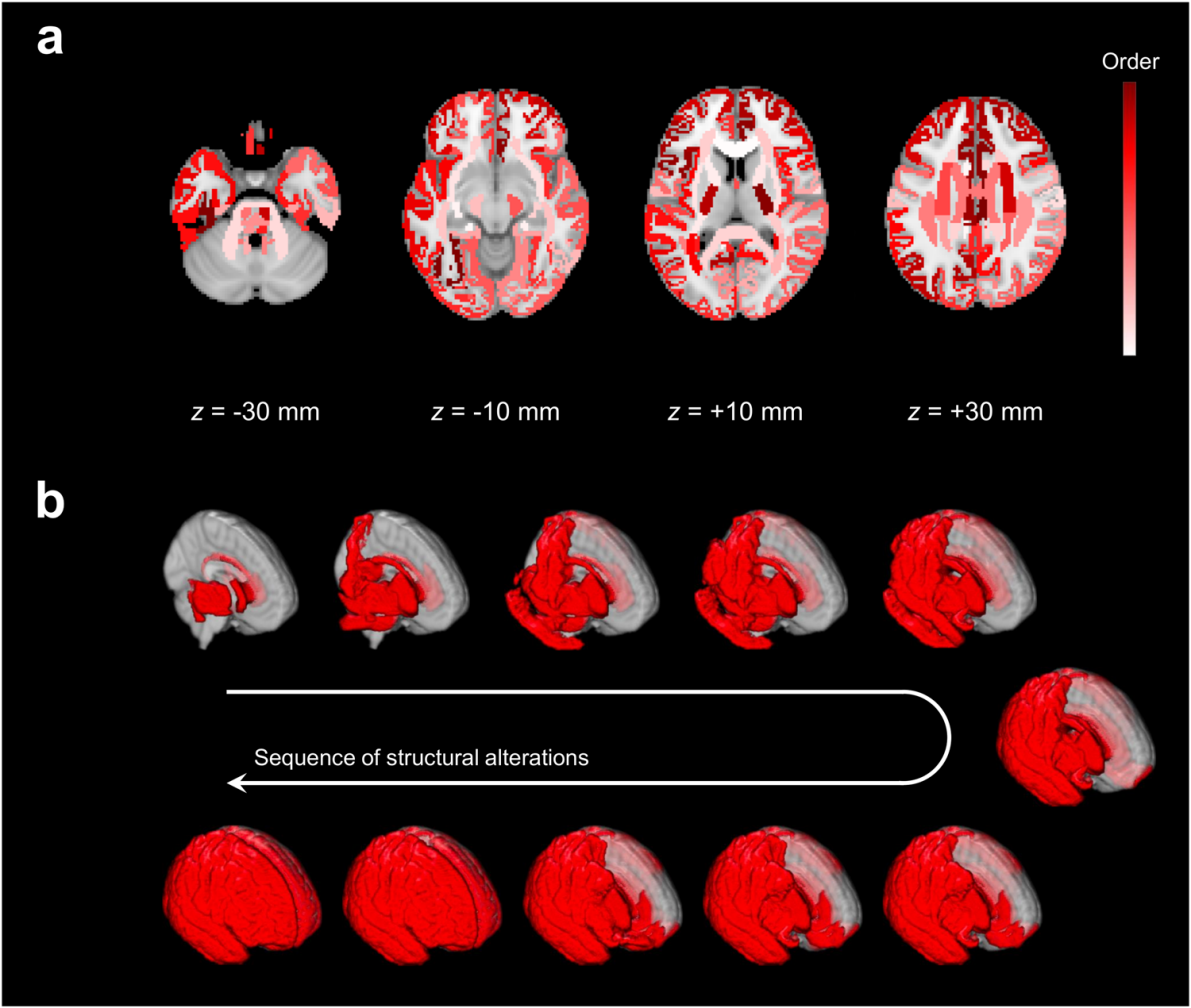
Ordering of structural alterations across 110 brain regions. **a** The earlier to later occurrence of structural alterations mapped with the white to maroon color scheme. **b** Snapshots of structural alterations displayed on a three dimensional rendered brain.

Comparisons of conditional probabilities for regional pair combinations between 110 brain regions, including 62 cortical GM regions and 48 WM regions, were significant for 14% (1643/11990) of all regional pair combinations (Supplementary Fig. 2). Figure 3 and Supplementary Table 2 display the ordering of structural alterations across 110 brain regions, as inferred by the sequence determined from one million Markov chain Monte Carlo trajectories. Although there were variances at each step of simulated sequences of structural alterations, as depicted in Supplementary Fig. 4, limited off-diagonal variances tended toward strong confidence in the ordering.

Consistent to the ordering between cortical GM regions and WM regions, the sequence of structural alterations described the earlier occurrence of structural alterations in most of WM regions than in cortical GM regions. According to the determined sequence, decreased integrity in WM regions initiated at commissural tracts, such as the corpus callosum, and spread through association tracts, such as the cingulum, fronto-occipital fasciculus, superior longitudinal fasciculus, and uncinate fasciculus, to projection tracts, such as the corticospinal tract. Reduced thickness in cortical GM regions started from sensory–motor cortices and spread through association cortices, comprising the parietal, temporal, and occipital cortices, to the prefrontal cortex. Of note, although there were variations in the sequence of structural alterations, the primary findings were consistently reproduced for different cutoff values to determine structural alterations (Supplementary Fig. 5 and Supplementary Table 3) and a different atlas to parcellate cortical GM regions (Supplementary Fig. 6 and Supplementary Table 4).

### Staging of structural alterations according to clinical status

**Fig. 4:**
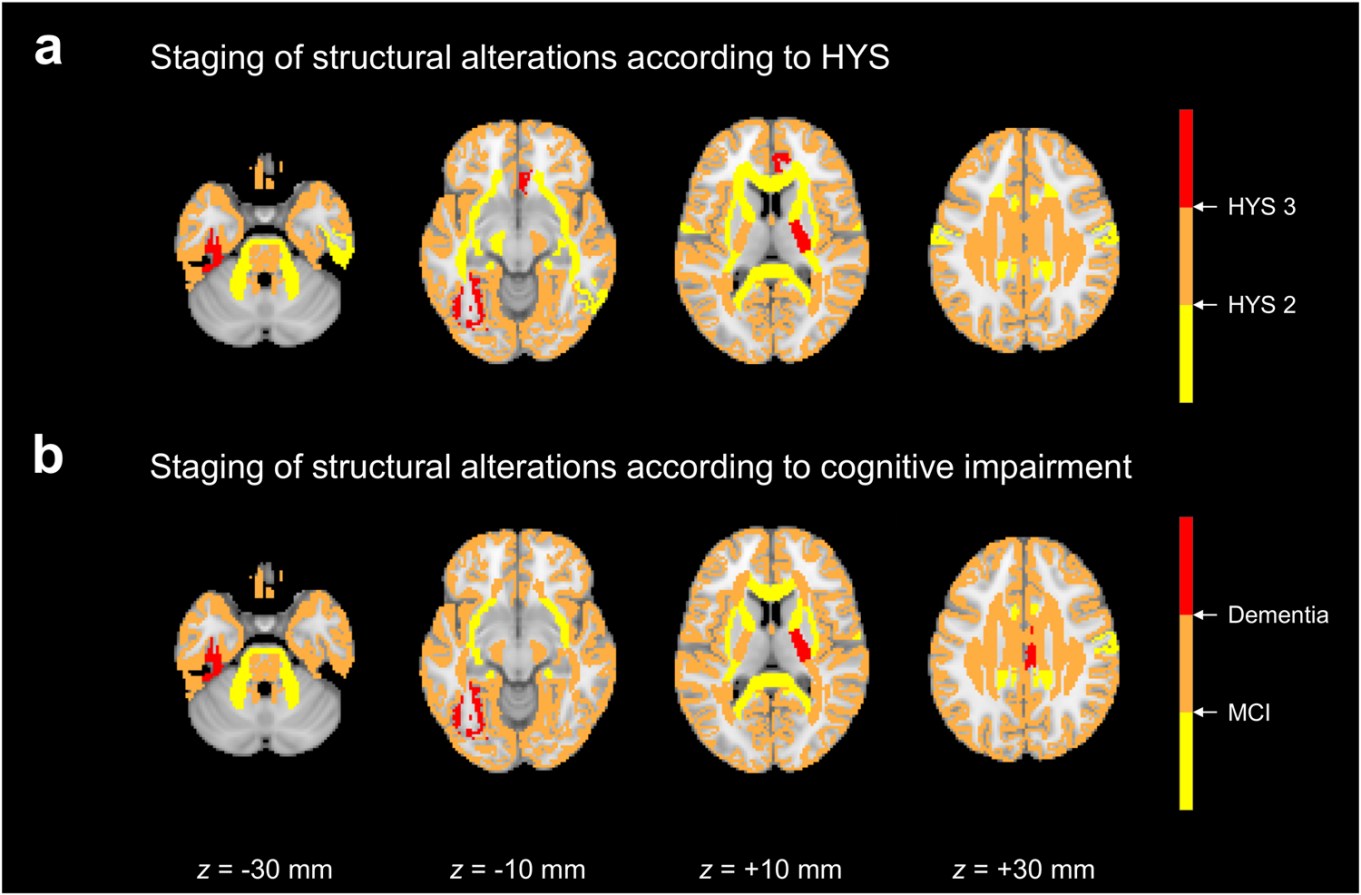
Staging of structural alterations according to changes in clinical status. **a** Structural alterations staged according to the progression of Hoehn and Yahr Stage (HYS) from <2, via ≥2 and <3, to ≥3. **b** Structural alterations staged according to the advancement of cognitive impairment from normal cognition, via mild cognitive impairment (MCI), to dementia.

The sequence of structural alterations could be staged according to the progression of HYS to 2 and 3 or the development of MCI and dementia (Fig. 4). With advancing HYS, among 110 brain regions, structural alterations were observed in 23 brain regions before reaching HYS 2 and in additional 84 and 3 brain regions after reaching HYS 2 and 3, respectively (Supplementary Table 5). With progressive cognitive impairment, among 110 brain regions, structural alterations were manifested in 14 brain regions before the incidence of cognitive impairment and in additional 93 and 3 brain regions after the development of MCI and dementia, respectively (Supplementary Table 5). According to the determined stages, structural alterations occurring prior to the development of HYS 2 or MCI were mostly seen in WM regions.

### Association between disease states and symptom severity

Considering that the sequence of structural alterations inferred for the patient cohort implied 110 disease states corresponding to different extents of structural alterations across 110 brain regions, individual patients’ disease states were predicted as those that best described observed structural alterations among the 110 disease states. The Kendall rank correlation between disease states predicted by the sequence of structural alterations and the severity of clinical symptoms in terms of the total score on the UPDRS was significant (Kendall’s *τ* = 0.11, *P* value = 0.04) toward the ordinal association of later disease states with more severe clinical symptoms.

## Discussion

MRI markers of brain structural alterations, as a surrogate for pathological markers, appear to reflect the progression of PD, while the ordering of brain structural alterations remains unclear. In this study, we adopted the notion of event-based disease progression modeling and applied stochastic simulation to infer the ordering of structural alterations in terms of cortical thinning and WM disintegrity in the brain with PD. Based on individual brain regions’ probabilities of structural alterations occurring at specific steps of a sequence, we inferred the ordering of structural alterations across brain regions. We demonstrated that the sequence of structural alterations could represent PD progression according to advancing Hoehn and Yahr stages or progressive cognitive impairment and, furthermore, that individual patients’ disease states predicted by the sequence of structural alterations could relate to the worsening of clinical symptoms.

While determining the temporal precedence of structural alterations directly through cross-sectional observations tends to be inconclusive, the presence of WM structural alterations in the absence or limited presence of GM structural alterations in cross-sectional observations^19,20^ may indicate the earlier occurrence of structural alterations in the WM than in the GM. Indeed here we showed that WM structural alterations could be generally ahead of GM structural alterations when we only included cortical GM regions, indicating consistency with the pathological development of cortical Lewy bodies that could be preceded by axonal transport blockage^21^. Considering the occurrence of structural alterations in subcortical GM regions but not in cortical GM regions at the early stage of PD^22^, the ordering of structural alterations between subcortical GM regions and WM regions might be more comparable than was observed in this study.

According to the sequence of brain structural alterations, decreased integrity in WM regions started to happen in the corpus callosum, cingulum, and internal and external capsules before the occurrence of reduced thickness in cortical GM regions and eventually spread to the corticospinal tract. Indeed, cross-sectional observations at the early stage of PD displayed structural alterations across extensive WM tracts^23^, but the sparing of the corticospinal tract^24,25^. The current finding that WM disintegrity tended to occur earlier in rostral regions than in caudal regions of the tracts, that is, the genu than the splenium in the corpus callosum, the anterior limb than the posterior limb in the internal capsule, and the anterior corona radiata than the posterior corona radiata, may represent earlier disintegrity in the WM closer to the mesostriatal or nigrostriatal system.

Besides, the sequence of brain structural alterations revealed that reduced thickness in cortical GM regions spread from sensory–motor and association cortices. The earlier occurrence of structural alterations in sensory–motor cortices than in other cortical GM regions has been often observed^7,26,27^, which might be resulted from preferential impairment of the motor loop of cortico-basal ganglia connections in PD^28^. While there were controversies about the ordering of structural alterations between anterior and posterior cortical GM regions, here we showed a trend for the earlier involvement of association cortices than the prefrontal cortex in cortical thinning. Considering that cortical thinning in the prefrontal cortex tended to occur earlier in ventral regions than in dorsal regions, cortical thinning in ventral regions of the prefrontal cortex may not always be behind that in association cortices^27^. The earlier involvement of ventral regions than dorsal regions of the prefrontal cortex in cortical thinning may be related to impaired dopaminergic projections to the prefrontal cortex or olfactory dysfunction^29^.

Clinical interests would be specifically in the relevance of the ordering of structural alterations to the severity of clinical symptoms. At the patient cohort level, we proposed the staging of structural alterations according to the progression of HYS or the advancement of cognitive impairment. While WM structural alterations could occur even before the incidence of bilateral symptoms/signs or MCI, structural alterations occurring after the development of postural instability or dementia may be limited, indicating that most of structural alterations could be displayed before major disease complications in PD. In addition, at the individual patient level, given disease states predicted from the sequence of structural alterations, we demonstrated the association of later disease states with more severe clinical symptoms, suggesting the clinical feasibility of predicting individual patients’ disease states based on the progression of PD.

While there were few studies that have directly explored the ordering of structural alterations in the brain with PD, here we inferred the ordering of structural alterations by employing stochastic simulation on top of event-based disease progression modeling. However, the ordering of structural alterations is not yet on a completely sound footing, exposing several limitations. First, as temporal precedence in the ordering was established based on cross-sectional observations, the ordering would warrant validation through longitudinal follow-up. In addition, the assumption of consistency in the ordering according to progressive symptoms may not fully reflect reality in which the reversion as well as progression of symptoms could be seen^30^ and prognosis could be different between distinct clinical subtypes of PD^31^, so that the assumption needs to be further developed.

Considering that we have modeled the probabilistic relationship of structural alterations between brain regions by conditional probability, our model may be regarded as having a similarity to a probabilistic graphical model such as a Bayesian belief network. Indeed, both models comprise variables and conditional relationships between them, which could be represented as nodes and edges, respectively, in a graph. Whereas a Bayesian belief network can deal with various directed acyclic graphs, our model focuses on a serial graph corresponding to a series of discrete events, primarily aiming to estimate the structure of the graph representing the sequence of the events. Although our model is believed to have worked well under the notion of event-based disease progression modeling, it could be extended to a Bayesian belief network to be able to describe diverse disease progression scenarios.

To summarize, we showed that cross-sectional phenotypic observations of the pathological brain with MRI could be employed to identify the progression of PD via the ordering of brain structural alterations, which was inferred by stochastic simulation based on the probabilistic relationship of structural alterations between brain regions. We are planning future work motivated to fill gaps between the propagation of brain structural alterations and the spreading of Lewy body pathology^32^. This study would be a step toward identifying PD progression, which would lead to a better understanding of progressive clinical symptoms and therefore to an improved diagnosis and prognosis about clinical status.

## Methods

### Study participants

In this retrospective study, among a total of 167 consecutive patients with PD who visited the clinic between November 2018 and November 2019, 130 patients with a disease onset beyond 40 years of age who underwent clinical and neuropsychological evaluation and MRI scanning were included, whereas the other 37 patients were excluded due to incomplete data or an excessive interval between neuropsychological evaluation and MRI scanning (Supplementary Fig. 7). The clinical diagnosis of PD was made according to the diagnostic criteria of the UK PD Brain Bank^33^. As age- and sex-matched normative controls, 75 healthy individuals (60 ± 11 years, 49 men) whose MRI scans were available were selected from the Parkinson’s Progression Markers Initiative (https://www.ppmi-info.org/). The study was approved by the institutional review board at the Seoul St. Mary’s Hospital, Seoul, Korea, and the obtaining of written informed consent was exempted from the institutional review board due to the retrospective nature of the study.

### Clinical and neuropsychological evaluation

As rating scales of PD, the modified Hoehn and Yahr Staging Scale were used to assess the stage of PD, and the UPDRS was used to evaluate the severity of motor and non-motor symptoms. In addition, the Mini-Mental State Examination and Geriatric Depression Scale were assessed, and medications were evaluated by calculating the levodopa equivalent daily dose^34^.

Cognitive status with respect to the presence and severity of cognitive impairment was evaluated using the Seoul Neuropsychological Screening Battery that comprises comprehensive neuropsychological tests in five cognitive domains. Specifically. attention was assessed with the Digit Span Test, memory with the Seoul Verbal Learning Test and Rey–Osterrieth Complex Figure Test, language with the Boston Naming Test, visuospatial function with the Rey–Osterrieth Complex Figure Test, and executive function with the Controlled Oral Word Association Test. An adjusted *z*-score with respect to age, sex, and years of education was computed for each neuropsychological test, and a negative *z*-score below −1, that is, below one standard deviation from the mean of the normative distribution, was considered indicative of cognitive impairment in terms of the neuropsychological test. A domain *z*-score was acquired as the average of *z*-scores over constituent neuropsychological tests, and an aggregate *z*-score for each patient was obtained as the average of domain *z*-scores over the five domains.

Through the neuropsychological tests, the patients with PD were classified as PD-NC, PD-MCI, or PD-D. PD-MCI was diagnosed according to the criteria for MCI in PD level I as suggested by the Movement Disorder Society Task Force^35^, and PD-D was diagnosed according to the criteria for probable dementia in PD as developed by the Movement Disorder Society Task Force^36^. In more detail, when significant impairment on daily life caused by cognitive deficit was supposed for patients with scores ≥ 1 on the Clinical Dementia Rating scale or ≥0.35 on the Instrumental Activities of Daily Living scale, PD-MCI applied to cognitive impairment in at least two neuropsychological tests within a single domain or across different domains without impairment on daily life, whereas PD-D applied to cognitive impairment in at least two of the four core domains, including attention, memory, visuospatial function, and executive function, with impairment on daily life.

### MRI data acquisition

MRI data, including structural MRI and diffusion-weighted MRI, were collected using an Ingenia 3T MRI system (Philips Healthcare, Best, Netherlands) for the patients and site-dependent MRI systems of different manufacturers for the normative controls. For the patients, structural MRI data composed of one volume image in sagittal planes were acquired with the three-dimensional T1 turbo field echo sequence: number of slices = 176, slice thickness = 1.0 mm, matrix size = 256 × 256, and in-plane resolution = 1.0 mm × 1.0 mm; and diffusion-weighted MRI data composed of 33 volume images in axial planes, including 32 with diffusion weighting at *b* value = 1000 s/mm^2^ and one without diffusion weighting, were acquired with the diffusion tensor imaging sequence: number of slices = 72, slice thickness = 2.0 mm, matrix size = 128 × 128, and in-plane resolution = 1.8 mm × 1.8 mm. For the normative controls, structural MRI data consisted of one volume image in sagittal planes: number of slices = 176, slice thickness = 1.0 mm, matrix size = 240 × 256, and in-plane resolution = 1.0 mm × 1.0 mm; and diffusion-weighted MRI data consisted of 65 volume images in axial planes, comprising 64 with diffusion weighting at *b* value = 1000 s/mm^2^ and one without diffusion weighting: number of slices = 72, slice thickness = 2.0 mm, matrix size = 116 × 116, and in-plane resolution = 2.0 mm × 2.0 mm.

### MRI data analysis

Tools in CIVET (https://mcin.ca/technology/civet/) were used for cortical morphometric analysis of structural MRI data. The brain was classified into different tissue types, and then hemispheric cortical surfaces were reconstructed. The average of vertex-wise cortical thickness values was assigned to each of 62 cortical GM regions (Supplementary Table 6) parcellated according to the Desikan–Killiany–Tourville atlas^37^. Tools in FSL (http://fsl.fmrib.ox.ac.uk/fsl/) were used for diffusion tensor analysis of diffusion-weighted MRI data. After correction for distortions induced by eddy currents and simple head motion, a diffusion tensor was modeled at each voxel to compute fractional anisotropy (FA) that reflects the integrity of WM. In addition, the tract-based spatial statistics approach^38^ was applied to alleviate partial volume effects, such that FA values were projected onto an alignment-invariant tract representation. The average of voxel-wise FA values was assigned to each of 48 WM regions (Supplementary Table 6) parcellated according to the ICBM DTI-81 atlas^39^. Throughout the process, quality control was assured by assessing outcomes at each step by visual inspection.

### Conditional probability of structural alterations

In accordance with a large body of evidence, we assumed that a reduction in the thickness of a cortical GM region, that is, cortical thinning, and a decrease in the FA of a WM region, that is, WM disintegrity, could reflect a structural alteration of the respective brain region. For each patient, structural alterations in individual brain regions were evaluated in terms of the deviation of the patient’s value of cortical thickness or FA from the mean of the normative controls’ values, with all values adjusted for age and sex. Specifically, structural alterations were quantified as weighted values between 0 (at the mean of the normative controls’ values) and 1 (at or below 2.5 standard deviations from the mean of the normative controls’ values).

Conditional probability is concerned with the probability of an event occurring, given that another event has occurred, so that it may be used to describe the sequential order of the events. In the context of disease progression, given the presence and absence of abnormalities in two brain regions, *X* and *Y*, the sequence of abnormalities between the two brain regions can be estimated based on two conditional probabilities, *P*(*X*+| *Y*−) and *P*(*Y*+| *X*−)^14^. Events of abnormalities may be defined by detecting pathological agents^14,40^ or by assessing brain structural alterations as performed in this study, providing probabilities of the presence and absence of abnormalities, *P*(*X*+) and *P*(*X*−), for every brain region, *X*, and conditional probabilities, *P*(*X*+| *Y*−) and *P*(*Y*+| *X*−), between every pair of brain regions, *X* and *Y*: *P*(*X*+| *Y*−) = *P*(*X*+∩ *Y*−)/*P*(*Y*−) and *P*(*Y*+| *X*−) = *P*(*Y*+∩ *X*−)/*P*(*X*−).

### Ordering between cortical thinning and WM disintegrity

For each regional pair combination of a cortical GM region, *X*, and a WM region, *Y*, the ordering between cortical thinning and WM disintegrity was estimated by comparing *P*(*X*+| *Y*−) and *P*(*Y*+| *X*−) using the McNemar’s test^41^, in which the null hypothesis of marginal homogeneity stated that both conditional probabilities were equally likely. Statistical significance was determined at a *P* value ≤ 0.05 corrected for multiple comparisons using a false discovery rate method.

In addition, for a specific cortical GM region, *X*, *P*(*X*+| *Y*−) was averaged over 48 WM regions, *Y*’s, to assess a tendency towards the precedence of thinning in the cortical GM region over WM disintegrity, collectively stated as *P*(GM+| WM−). Similarly, for a specific WM region, *Y*, *P*(*Y*+| *X*−) was averaged over 62 cortical GM regions, *X*’s, to assess a tendency towards the precedence of disintegrity in the WM region over cortical thinning, collectively stated as *P*(WM+| GM−).

### Ordering of structural alterations across all brain regions

Following underlying assumptions of event-based disease progression modeling^10–12^, we supposed that the ordering of structural alterations could represent a monotonic progression and, additionally, we regarded the progression as being stochastic. Thus, the ordering of structural alterations between 110 brain regions, including 62 cortical GM regions and 48 WM regions, was inferred by simulating the stochastic progression of structural alternation events across the brain regions by adopting a Markov chain Monte Carlo method. The probability of a structural alteration occurring at a specific step of a sequence was computed by *P*(*X*+| ⋯, *Y*−, ⋯) for a brain region, *X*, and other brain regions, ⋯, *Y*, ⋯, structural alterations of which not occurring yet at the step of the sequence. Considering variability in the spreading of structural alterations, which brain region would show a structural alteration at a specific step was determined via random sampling according to the individual brain regions’ probabilities of structural alterations at the step. From one million Markov chain Monte Carlo trajectories of structural alteration events, the ordering was finally determined from the sequence composed of a series of brain regions structural alterations of which occurred most frequently at respective steps of simulated sequences.

Considering arbitrariness to the choice of a specific cutoff value to determine structural alterations and the choice of a particular atlas to parcellate brain regions, we examined the influences of different choices of them on the ordering of structural alterations. Simulations of stochastic progression were repeatedly carried out in the same way to assess possible variations in the sequence of structural alterations in cases of structural alterations determined based on the cutoff of 2.0 or 3.0 standard deviations from the mean of the normative controls’ values and cortical GM regions parcellated according to the AAL atlas^42^.

### Staging of structural alterations according to clinical status

Simulations of stochastic progression were additionally conducted by including clinical events together with structural alternation events to stage the sequence of structural alterations according to changes in clinical status. The progression of HYS to 2 and 3 was added as clinical events in one simulation, while the advancement of cognitive impairment to MCI and dementia was added as clinical events in another simulation. For both simulations, the ordering was determined from one million Markov chain Monte Carlo trajectories of clinical events and structural alteration events, and structural alteration events that occurred between two adjacent clinical events were grouped into the same status.

### Association between disease states and symptom severity

By adopting the notion of using the scalar product to evaluate pattern similarity, the sum of the entry-wise product between 110 entries indicating observed structural alterations across 110 brain regions for a patient and another 110 entries representing expected structural alterations across 110 brain regions according to each disease state (for instance, [1, 0, 0, ⋯, 0] for the first disease state, [1, 1, 0, ⋯, 0] for the second disease state, and so on) was computed, and the disease state for which the scalar product was maximized was selected as the patient’s disease state. For the patients, the ordinal association between disease states predicted by the sequence of structural alterations and the severity of clinical symptoms evaluated with the UPDRS was assessed by computing a Kendall rank correlation. Statistical significance was determined at a *P* value ≤ 0.05.

## Supplementary information

Supplementary Information

## Data Availability

Part of the data used in this study was obtained from the Parkinson’s Progression Markers Initiative (https://www.ppmi-info.org/). The other data that support the findings of this study are available from the corresponding author upon reasonable request.
