# Supplementary material for: Simulating the progression of brain structural alterations in Parkinson’s disease
Source: NPJ Parkinsons Dis. 2022 Jun 28;8:86. doi: 10.1038/s41531-022-00349-0 (PMC9240031; doi:10.1038/s41531-022-00349-0)
Supplement: Supplementary file 1 — Supplementary Information [file 41531_2022_349_MOESM1_ESM.pdf]

Supplementary Information:

Simulating the Progression of Brain Structural Alterations in Parkinson Disease

Chang-hyun Park[1,2], Na-Young Shin[1], Sang-Won Yoo[3], Haeseok Seo[4], Uicheul Yoon[4], Ji-Yeon Yoo[3], Kookjin Ahn[1], and Joong-Seok Kim[3]

[1]Department of Radiology, College of Medicine, Catholic University of Korea, Seoul, Korea
[2]Center for Neuroprosthetics and Brain Mind Institute, Swiss Federal Institute of Technology (EPFL), Geneva, Switzerland
[3]Department of Neurology, College of Medicine, Catholic University of Korea, Seoul, Korea
[4]Department of Biomedical Engineering, College of Bio and Medical Sciences, Daegu Catholic University, Gyeongsan, Gyeongbuk, Korea

Contents:

Supplementary Tables 1-6
Supplementary Figures 1-7

Supplementary Table 1. Ordering of structural alterations between cortical grey matter (GM) regions and white matter (WM) regions. In **a**, cortical GM regions are ordered by *P*(GM+|WM−) − *P*(WM+|GM−), and in **b**, WM regions are ordered by *P*(WM+|GM−) − *P*(GM+|WM−).

(**a**)

Order	Cortical GM region	***P*(GM+|WM−) − *P*(WM+|GM−)**	***P*(GM+|WM−)**	***P*(WM+|GM−)**
1	Right postcentral	0.15	0.69	0.54
2	Left postcentral	0.07	0.57	0.50
3	Right inferior temporal	0.05	0.57	0.53
4	Right precentral	0.03	0.52	0.49
5	Right pericalcarine	0.02	0.53	0.52
6	Right transverse temporal	0.01	0.48	0.47
7	Left parahippocampal	0.00	0.51	0.51
8	Right supramarginal	-0.01	0.48	0.49
9	Right parahippocampal	-0.01	0.49	0.50
10	Right cuneus	-0.01	0.48	0.50
11	Left pericalcarine	-0.02	0.48	0.50
12	Right lingual	-0.02	0.48	0.50
13	Left precentral	-0.03	0.44	0.47
14	Right pars orbitalis	-0.03	0.44	0.48
15	Right superior temporal	-0.04	0.45	0.49
16	Right superior parietal	-0.05	0.42	0.47
17	Right lateral occipital	-0.06	0.43	0.49
18	Left lingual	-0.07	0.41	0.48
19	Right middle temporal	-0.07	0.43	0.50
20	Right inferior parietal	-0.07	0.40	0.48
21	Left lateral orbitofrontal	-0.07	0.41	0.48
22	Left superior parietal	-0.09	0.36	0.45
23	Left transverse temporal	-0.09	0.36	0.45
24	Left caudal middle frontal	-0.11	0.33	0.44
25	Right caudal middle frontal	-0.11	0.34	0.45
26	Left medial orbitofrontal	-0.12	0.34	0.46
27	Left lateral occipital	-0.12	0.33	0.45
28	Left rostral anterior cingulate	-0.12	0.35	0.47
29	Right fusiform	-0.13	0.34	0.47
30	Left isthmus cingulate	-0.13	0.33	0.46

31	Right isthmus cingulate	-0.14	0.33	0.46
32	Right paracentral	-0.14	0.31	0.45
33	Right insula	-0.14	0.33	0.47
34	Left superior temporal	-0.15	0.30	0.45
35	Right entorhinal	-0.15	0.32	0.47
36	Left cuneus	-0.15	0.31	0.46
37	Left inferior temporal	-0.16	0.29	0.45
38	Right precuneus	-0.17	0.28	0.45
39	Left supramarginal	-0.17	0.26	0.44
40	Right pars opercularis	-0.18	0.27	0.45
41	Left entorhinal	-0.18	0.26	0.45
42	Left insula	-0.18	0.27	0.45
43	Left middle temporal	-0.19	0.26	0.45
44	Left pars orbitalis	-0.20	0.24	0.44
45	Right pars triangularis	-0.20	0.23	0.43
46	Right lateral orbitofrontal	-0.21	0.24	0.44
47	Left rostral middle frontal	-0.21	0.23	0.44
48	Left superior frontal	-0.21	0.22	0.43
49	Left inferior parietal	-0.22	0.22	0.44
50	Right rostral middle frontal	-0.22	0.21	0.44
51	Right medial orbitofrontal	-0.23	0.21	0.44
52	Left precuneus	-0.23	0.20	0.44
53	Right superior frontal	-0.24	0.19	0.43
54	Left posterior cingulate	-0.24	0.19	0.43
55	Left pars triangularis	-0.24	0.18	0.43
56	Left paracentral	-0.25	0.18	0.43
57	Left caudal anterior cingulate	-0.27	0.16	0.43
58	Left pars opercularis	-0.28	0.14	0.42
59	Right rostral anterior cingulate	-0.29	0.14	0.43
60	Right posterior cingulate	-0.30	0.12	0.42
61	Right caudal anterior cingulate	-0.30	0.12	0.42
62	Left fusiform	-0.33	0.09	0.42

(b)

**Order**	**WM region**	***P*(WM+|GM−) − *P*(GM+|WM−)**	***P*(WM+|GM−)**	***P*(GM+|WM−)**
1	Genu of corpus callosum	0.38	0.80	0.42

2	Left cingulum (cingulate gyrus)	0.34	0.71	0.38
3	Anterior limb of left internal capsule	0.32	0.68	0.37
4	Left fornix / stria terminalis	0.32	0.70	0.38
5	Left cingulum (hippocampus)	0.30	0.72	0.43
6	Anterior limb of right internal capsule	0.29	0.66	0.37
7	Right cingulum (hippocampus)	0.29	0.69	0.40
8	Retrolenticular part of left internal capsule	0.29	0.66	0.37
9	Left external capsule	0.28	0.65	0.37
10	Right external capsule	0.27	0.64	0.38
11	Splenium of corpus callosum	0.25	0.60	0.35
12	Middle cerebellar peduncle	0.25	0.61	0.36
13	Left anterior corona radiata	0.24	0.59	0.35
14	Right cingulum (cingulate gyrus)	0.24	0.60	0.36
15	Right anterior corona radiata	0.23	0.57	0.34
16	Right fornix / stria terminalis	0.22	0.59	0.37
17	Retrolenticular part of right internal capsule	0.21	0.58	0.37
18	Right sagittal stratum	0.18	0.53	0.35
19	Left sagittal stratum	0.18	0.54	0.36
20	Left cerebral peduncle	0.18	0.52	0.34
21	Right posterior thalamic radiation	0.14	0.48	0.34
22	Left superior cerebellar peduncle	0.14	0.48	0.34
23	Left superior fronto-occipital fasciculus	0.14	0.48	0.34
24	Left posterior corona radiata	0.11	0.43	0.32
25	Left medial lemniscus	0.10	0.42	0.31
26	Right superior longitudinal fasciculus	0.10	0.42	0.32
27	Right superior fronto-occipital fasciculus	0.10	0.43	0.33
28	Right superior cerebellar peduncle	0.09	0.41	0.32
29	Pontine crossing tract	0.09	0.41	0.32
30	Body of corpus callosum	0.09	0.39	0.30
31	Left superior longitudinal fasciculus	0.09	0.40	0.31
32	Right inferior cerebellar peduncle	0.08	0.39	0.31
33	Left inferior cerebellar peduncle	0.08	0.39	0.31
34	Left corticospinal tract	0.07	0.38	0.32
35	Right uncinate fasciculus	0.05	0.35	0.30
36	Right posterior corona radiata	0.05	0.36	0.31
37	Right cerebral peduncle	0.04	0.35	0.31

38	Fornix	0.03	0.32	0.29
39	Left superior corona radiata	0.02	0.31	0.29
40	Left uncinate fasciculus	0.00	0.29	0.29
41	Left tapetum	-0.02	0.25	0.27
42	Right medial lemniscus	-0.02	0.27	0.29
43	Left posterior thalamic radiation	-0.06	0.23	0.28
44	Right tapetum	-0.06	0.21	0.27
45	Right superior corona radiata	-0.09	0.19	0.28
46	Right corticospinal tract	-0.09	0.18	0.27
47	Posterior limb of left internal capsule	-0.10	0.17	0.27
48	Posterior limb of right internal capsule	-0.18	0.08	0.26

Supplementary Table 2. Ordering of structural alterations across 110 brain regions. Frequency means the relative frequency of a specific brain region's structural alteration occurring at the respective step of simulated sequences. P(X+|Y−) represents the probability of a specific brain region's structural alteration occurring earlier, whereas P(Y+|X−) expresses the probability of a specific brain region's structural alteration occurring later.

Order	Tissue	Brain region	Frequency	*P*(X+|Y−)	*P*(Y+|X−)
1	WM	Genu of corpus callosum	1.00	0.80	0.46
2	WM	Left cingulum (hippocampus)	0.95	0.74	0.49
3	WM	Left cingulum (cingulate gyrus)	0.66	0.71	0.43
4	WM	Left fornix / stria terminalis	0.49	0.70	0.44
5	WM	Right cingulum (hippocampus)	0.62	0.70	0.46
6	WM	Anterior limb of left internal capsule	0.80	0.68	0.41
7	WM	Retrolenticular part of left internal capsule	0.55	0.67	0.43
8	WM	Anterior limb of right internal capsule	0.48	0.66	0.42
9	WM	Left external capsule	0.51	0.65	0.42
10	WM	Right external capsule	0.60	0.64	0.43
11	GM	Right postcentral	0.77	0.64	0.38
12	WM	Middle cerebellar peduncle	0.65	0.62	0.42
13	WM	Right cingulum (cingulate gyrus)	0.44	0.61	0.43
14	WM	Splenium of corpus callosum	0.43	0.60	0.40
15	WM	Right fornix / stria terminalis	0.38	0.59	0.43
16	WM	Left anterior corona radiata	0.46	0.59	0.40
17	WM	Retrolenticular part of right internal capsule	0.65	0.58	0.42
18	WM	Right anterior corona radiata	0.87	0.56	0.39
19	WM	Left sagittal stratum	0.79	0.54	0.41
20	WM	Right sagittal stratum	0.71	0.53	0.40
21	WM	Left cerebral peduncle	0.82	0.52	0.39
22	GM	Left postcentral	0.68	0.52	0.36
23	GM	Right inferior temporal	0.69	0.52	0.39
24	WM	Right posterior thalamic radiation	0.44	0.48	0.39
25	WM	Left superior fronto-occipital fasciculus	0.31	0.48	0.39
26	WM	Left superior cerebellar peduncle	0.35	0.48	0.39
27	GM	Right pericalcarine	0.47	0.48	0.40
28	GM	Right precentral	0.69	0.47	0.37
29	GM	Left parahippocampal	0.73	0.46	0.38
30	GM	Right parahippocampal	0.20	0.44	0.38
31	WM	Right superior fronto-occipital fasciculus	0.19	0.43	0.39

32	WM	Left posterior corona radiata	0.18	0.43	0.37
33	GM	Right transverse temporal	0.16	0.44	0.35
34	WM	Right superior longitudinal fasciculus	0.12	0.42	0.38
35	WM	Left medial lemniscus	0.14	0.42	0.38
36	WM	Pontine crossing tract	0.14	0.43	0.39
37	GM	Right supramarginal	0.13	0.43	0.37
38	GM	Left pericalcarine	0.13	0.43	0.37
39	GM	Right lingual	0.15	0.43	0.38
40	GM	Right cuneus	0.17	0.43	0.37
41	WM	Right superior cerebellar peduncle	0.21	0.41	0.38
42	WM	Left superior longitudinal fasciculus	0.34	0.40	0.37
43	WM	Right inferior cerebellar peduncle	0.19	0.40	0.38
44	WM	Body of corpus callosum	0.18	0.38	0.36
45	WM	Left inferior cerebellar peduncle	0.19	0.39	0.37
46	WM	Left corticospinal tract	0.18	0.40	0.39
47	GM	Right pars orbitalis	0.17	0.40	0.37
48	GM	Left precentral	0.46	0.39	0.34
49	GM	Right superior temporal	0.41	0.39	0.36
50	GM	Right superior parietal	0.27	0.37	0.35
51	WM	Right uncinate fasciculus	0.20	0.36	0.37
52	GM	Right lateral occipital	0.19	0.38	0.37
53	WM	Right posterior corona radiata	0.17	0.35	0.36
54	GM	Left lingual	0.16	0.36	0.36
55	WM	Right cerebral peduncle	0.16	0.35	0.37
56	GM	Left lateral orbitofrontal	0.19	0.37	0.37
57	GM	Right middle temporal	0.23	0.37	0.38
58	GM	Right inferior parietal	0.45	0.36	0.36
59	WM	Fornix	0.69	0.34	0.37
60	WM	Left superior corona radiata	0.49	0.30	0.35
61	GM	Left transverse temporal	0.26	0.31	0.33
62	GM	Left superior parietal	0.26	0.31	0.33
63	WM	Left uncinate fasciculus	0.18	0.30	0.35
64	GM	Right caudal middle frontal	0.18	0.30	0.34
65	GM	Left caudal middle frontal	0.20	0.29	0.33
66	GM	Left medial orbitofrontal	0.17	0.30	0.35
67	GM	Left rostral anterior cingulate	0.19	0.31	0.36

68	GM	Right fusiform	0.17	0.30	0.36
69	GM	Left lateral occipital	0.18	0.29	0.34
70	GM	Right insula	0.16	0.29	0.36
71	GM	Right isthmus cingulate	0.16	0.29	0.36
72	GM	Left isthmus cingulate	0.17	0.29	0.35
73	GM	Right paracentral	0.18	0.27	0.33
74	WM	Right medial lemniscus	0.20	0.28	0.36
75	WM	Left tapetum	0.25	0.25	0.34
76	GM	Right entorhinal	0.25	0.28	0.36
77	GM	Left cuneus	0.25	0.26	0.35
78	GM	Left superior temporal	0.30	0.26	0.34
79	GM	Left inferior temporal	0.25	0.25	0.34
80	GM	Right precuneus	0.15	0.24	0.34
81	GM	Right pars opercularis	0.16	0.24	0.35
82	GM	Left supramarginal	0.15	0.23	0.33
83	WM	Left posterior thalamic radiation	0.14	0.22	0.34
84	GM	Left insula	0.14	0.24	0.35
85	GM	Left entorhinal	0.16	0.23	0.35
86	WM	Right tapetum	0.18	0.21	0.34
87	GM	Left middle temporal	0.17	0.22	0.34
88	GM	Right pars triangularis	0.15	0.20	0.34
89	GM	Left pars orbitalis	0.15	0.21	0.34
90	GM	Right lateral orbitofrontal	0.14	0.20	0.34
91	GM	Left rostral middle frontal	0.13	0.20	0.33
92	WM	Right superior corona radiata	0.12	0.19	0.34
93	GM	Left superior frontal	0.12	0.19	0.33
94	WM	Right corticospinal tract	0.11	0.20	0.35
95	GM	Right rostral middle frontal	0.12	0.18	0.34
96	GM	Left inferior parietal	0.13	0.18	0.33
97	GM	Right medial orbitofrontal	0.12	0.18	0.34
98	WM	Posterior limb of left internal capsule	0.11	0.17	0.34
99	GM	Left precuneus	0.13	0.17	0.33
100	GM	Right superior frontal	0.14	0.16	0.33
101	GM	Left pars triangularis	0.15	0.16	0.33
102	GM	Left paracentral	0.18	0.15	0.33
103	GM	Left posterior cingulate	0.15	0.17	0.34

104	GM	Left caudal anterior cingulate	0.23	0.14	0.34
105	GM	Left pars opercularis	0.22	0.12	0.33
106	GM	Right rostral anterior cingulate	0.17	0.12	0.34
107	GM	Right caudal anterior cingulate	0.18	0.11	0.33
108	WM	Posterior limb of right internal capsule	0.20	0.08	0.33
109	GM	Left fusiform	0.29	0.07	0.33
110	GM	Right posterior cingulate	0.14	0.11	0.33

GM, grey matter; and WM, white matter.

Supplementary Table 3. Ordering of structural alterations across 110 brain regions in the case of applying the cut-off of 2.0 (**a**) and 3.0 (**b**) standard deviations, instead of the default choice of 2.5 standard deviations, from the mean of normative controls' values in determining structural alterations in the brain regions.

(**a**)

Order	Tissue	Brain region
1	WM	Genu of corpus callosum
2	WM	Left cingulum (hippocampus)
3	WM	Right cingulum (hippocampus)
4	WM	Left cingulum (cingulate gyrus)
5	WM	Left fornix / stria terminalis
6	WM	Anterior limb of left internal capsule
7	WM	Retrolenticular part of left internal capsule
8	WM	Anterior limb of right internal capsule
9	WM	Left external capsule
10	WM	Right external capsule
11	WM	Middle cerebellar peduncle
12	WM	Right cingulum (cingulate gyrus)
13	WM	Right fornix / stria terminalis
14	WM	Splenium of corpus callosum
15	WM	Retrolenticular part of right internal capsule
16	WM	Left anterior corona radiata
17	WM	Right anterior corona radiata
18	WM	Left sagittal stratum
19	WM	Right sagittal stratum
20	GM	Right postcentral
21	WM	Left cerebral peduncle
22	WM	Left superior fronto-occipital fasciculus
23	WM	Right posterior thalamic radiation
24	WM	Left superior cerebellar peduncle
25	WM	Right superior fronto-occipital fasciculus
26	WM	Pontine crossing tract
27	WM	Right superior longitudinal fasciculus
28	WM	Left posterior corona radiata
29	WM	Left medial lemniscus
30	GM	Right inferior temporal
31	WM	Right superior cerebellar peduncle

32	WM	Left superior longitudinal fasciculus
33	WM	Left corticospinal tract
34	WM	Right inferior cerebellar peduncle
35	GM	Left postcentral
36	WM	Left inferior cerebellar peduncle
37	WM	Body of corpus callosum
38	GM	Left parahippocampal
39	GM	Right pericalcarine
40	GM	Right supramarginal
41	GM	Right parahippocampal
42	GM	Right precentral
43	WM	Right uncinate fasciculus
44	WM	Right cerebral peduncle
45	WM	Right posterior corona radiata
46	GM	Right transverse temporal
47	GM	Right lingual
48	GM	Left pericalcarine
49	GM	Right pars orbitalis
50	GM	Right cuneus
51	WM	Fornix
52	GM	Right superior parietal
53	GM	Right lateral occipital
54	WM	Left superior corona radiata
55	WM	Left uncinate fasciculus
56	GM	Right superior temporal
57	GM	Right inferior parietal
58	GM	Left lingual
59	GM	Right middle temporal
60	GM	Left precentral
61	GM	Left rostral anterior cingulate
62	GM	Right insula
63	WM	Right medial lemniscus
64	GM	Left lateral orbitofrontal
65	GM	Left caudal middle frontal
66	WM	Left tapetum
67	GM	Left transverse temporal

68	GM	Right caudal middle frontal
69	GM	Left superior parietal
70	GM	Right isthmus cingulate
71	GM	Right fusiform
72	GM	Left lateral occipital
73	GM	Left medial orbitofrontal
74	GM	Left isthmus cingulate
75	GM	Right entorhinal
76	WM	Left posterior thalamic radiation
77	GM	Right paracentral
78	GM	Left insula
79	WM	Right tapetum
80	GM	Right precuneus
81	GM	Left superior temporal
82	GM	Left cuneus
83	GM	Left inferior temporal
84	WM	Right superior corona radiata
85	GM	Right lateral orbitofrontal
86	WM	Right corticospinal tract
87	GM	Left entorhinal
88	GM	Left supramarginal
89	GM	Right pars opercularis
90	WM	Posterior limb of left internal capsule
91	GM	Left pars orbitalis
92	GM	Left middle temporal
93	GM	Right pars triangularis
94	GM	Right medial orbitofrontal
95	GM	Right rostral middle frontal
96	GM	Left inferior parietal
97	GM	Left rostral middle frontal
98	GM	Left superior frontal
99	GM	Right superior frontal
100	GM	Left paracentral
101	GM	Left precuneus
102	GM	Left pars triangularis
103	GM	Left posterior cingulate

104	GM	Left caudal anterior cingulate
105	GM	Right caudal anterior cingulate
106	GM	Right posterior cingulate
107	GM	Left pars opercularis
108	WM	Posterior limb of right internal capsule
109	GM	Left fusiform
110	GM	Right rostral anterior cingulate

(**b**)

**Order**	**Tissue**	**Brain region**
1	WM	Genu of corpus callosum
2	WM	Left cingulum (hippocampus)
3	WM	Left cingulum (cingulate gyrus)
4	WM	Left fornix / stria terminalis
5	WM	Right cingulum (hippocampus)
6	WM	Anterior limb of left internal capsule
7	WM	Anterior limb of right internal capsule
8	WM	Retrolenticular part of left internal capsule
9	WM	Left external capsule
10	WM	Right external capsule
11	WM	Middle cerebellar peduncle
12	WM	Splenium of corpus callosum
13	WM	Right cingulum (cingulate gyrus)
14	WM	Left anterior corona radiata
15	WM	Right fornix / stria terminalis
16	WM	Right anterior corona radiata
17	WM	Retrolenticular part of right internal capsule
18	GM	Right postcentral
19	WM	Left sagittal stratum
20	WM	Right sagittal stratum
21	WM	Left cerebral peduncle
22	WM	Right posterior thalamic radiation
23	WM	Left superior cerebellar peduncle
24	WM	Left superior fronto-occipital fasciculus
25	WM	Left posterior corona radiata
26	GM	Right inferior temporal
27	GM	Left postcentral

28	WM	Right superior fronto-occipital fasciculus
29	WM	Left medial lemniscus
30	WM	Right superior longitudinal fasciculus
31	WM	Right superior cerebellar peduncle
32	WM	Pontine crossing tract
33	WM	Left superior longitudinal fasciculus
34	WM	Body of corpus callosum
35	WM	Right inferior cerebellar peduncle
36	GM	Left parahippocampal
37	WM	Left inferior cerebellar peduncle
38	GM	Right transverse temporal
39	GM	Right pericalcarine
40	GM	Right precentral
41	GM	Right supramarginal
42	WM	Left corticospinal tract
43	GM	Right parahippocampal
44	WM	Right uncinate fasciculus
45	GM	Right lingual
46	WM	Right posterior corona radiata
47	GM	Right cuneus
48	GM	Left pericalcarine
49	GM	Right pars orbitalis
50	WM	Right cerebral peduncle
51	GM	Right superior parietal
52	WM	Fornix
53	GM	Left precentral
54	WM	Left superior corona radiata
55	GM	Right lateral occipital
56	GM	Right superior temporal
57	GM	Left lingual
58	GM	Right inferior parietal
59	WM	Left uncinate fasciculus
60	GM	Left caudal middle frontal
61	GM	Left rostral anterior cingulate
62	GM	Right middle temporal
63	GM	Left transverse temporal

64	GM	Right insula
65	GM	Left superior parietal
66	GM	Right caudal middle frontal
67	GM	Left lateral orbitofrontal
68	WM	Left tapetum
69	WM	Right medial lemniscus
70	GM	Left lateral occipital
71	GM	Right isthmus cingulate
72	GM	Left isthmus cingulate
73	GM	Left medial orbitofrontal
74	GM	Right paracentral
75	GM	Right fusiform
76	WM	Left posterior thalamic radiation
77	GM	Right entorhinal
78	GM	Right precuneus
79	GM	Left superior temporal
80	WM	Right tapetum
81	GM	Left insula
82	GM	Left inferior temporal
83	GM	Left cuneus
84	GM	Left supramarginal
85	GM	Right lateral orbitofrontal
86	GM	Left entorhinal
87	WM	Right superior corona radiata
88	WM	Right corticospinal tract
89	GM	Right pars opercularis
90	GM	Left pars orbitalis
91	GM	Left middle temporal
92	GM	Right pars triangularis
93	WM	Posterior limb of left internal capsule
94	GM	Right medial orbitofrontal
95	GM	Left superior frontal
96	GM	Left inferior parietal
97	GM	Left rostral middle frontal
98	GM	Right rostral middle frontal
99	GM	Right superior frontal

100	GM	Left precuneus
101	GM	Left paracentral
102	GM	Left pars triangularis
103	GM	Left posterior cingulate
104	GM	Left caudal anterior cingulate
105	GM	Right caudal anterior cingulate
106	GM	Left pars opercularis
107	GM	Right posterior cingulate
108	GM	Left fusiform
109	WM	Posterior limb of right internal capsule
110	GM	Right rostral anterior cingulate

GM, grey matter; and WM, white matter.

Supplementary Table 4. Ordering of structural alterations across 124 brain regions in the case of parcellating the brain into 76 cortical grey matter (GM) regions, instead of the default choice of 62 ones, and 48 white matter (WM) regions.

Order	Tissue	Brain region
1	WM	Genu of corpus callosum
2	WM	Left cingulum (hippocampus)
3	WM	Left cingulum (cingulate gyrus)
4	WM	Left fornix / stria terminalis
5	WM	Right cingulum (hippocampus)
6	WM	Anterior limb of left internal capsule
7	WM	Retrolenticular part of left internal capsule
8	WM	Anterior limb of right internal capsule
9	WM	Left external capsule
10	WM	Right external capsule
11	WM	Middle cerebellar peduncle
12	WM	Right cingulum (cingulate gyrus)
13	WM	Splenium of corpus callosum
14	WM	Right fornix / stria terminalis
15	WM	Left anterior corona radiata
16	WM	Retrolenticular part of right internal capsule
17	WM	Right anterior corona radiata
18	WM	Left sagittal stratum
19	GM	Right postcentral
20	WM	Right sagittal stratum
21	WM	Left cerebral peduncle
22	WM	Right posterior thalamic radiation
23	WM	Left superior cerebellar peduncle
24	WM	Left superior fronto-occipital fasciculus
25	GM	Right inferior temporal
26	WM	Left posterior corona radiata
27	WM	Right superior fronto-occipital fasciculus
28	WM	Right superior longitudinal fasciculus
29	WM	Left medial lemniscus
30	WM	Pontine crossing tract
31	GM	Left postcentral
32	WM	Right superior cerebellar peduncle

33	WM	Left superior longitudinal fasciculus
34	WM	Body of corpus callosum
35	WM	Right inferior cerebellar peduncle
36	WM	Left inferior cerebellar peduncle
37	GM	Right precentral
38	WM	Left corticospinal tract
39	GM	Right supramarginal
40	GM	Right calcarine fissure
41	GM	Right Heschl
42	WM	Right uncinate fasciculus
43	GM	Left parahippocampal
44	GM	Right parahippocampal
45	GM	Right lingual
46	WM	Right posterior corona radiata
47	WM	Right cerebral peduncle
48	GM	Left calcarine fissure
49	GM	Right angular
50	GM	Right cuneus
51	GM	Right superior parietal
52	GM	Left precentral
53	GM	Right superior temporal
54	WM	Fornix
55	WM	Left superior corona radiata
56	GM	Left superior frontal, orbital part
57	WM	Left uncinate fasciculus
58	GM	Left lingual
59	GM	Right inferior occipital
60	GM	Right inferior frontal, orbital part
61	GM	Right temporal pole (superior temporal)
62	GM	Left superior parietal
63	WM	Right medial lemniscus
64	GM	Left straight
65	GM	Right middle occipital
66	GM	Right superior occipital
67	WM	Left tapetum
68	GM	Right middle temporal

69	GM	Right paracentral lobule
70	GM	Right Rolandic operculum
71	GM	Right insula
72	GM	Left temporal pole (superior temporal)
73	GM	Right temporal pole (middle temporal)
74	GM	Right precuneus
75	GM	Right fusiform
76	GM	Left superior occipital
77	GM	Left olfactory
78	GM	Left paracentral lobule
79	GM	Left inferior temporal
80	WM	Left posterior thalamic radiation
81	GM	Left Heschl
82	GM	Left cuneus
83	GM	Left superior frontal, medial orbital
84	WM	Right tapetum
85	GM	Left superior temporal
86	GM	Right middle frontal, orbital part
87	GM	Left supramarginal
88	GM	Left anterior cingulate and paracingulate
89	GM	Right superior frontal, orbital part
90	GM	Left angular
91	GM	Left middle frontal
92	GM	Left insula
93	GM	Left superior frontal, dorsolateral
94	GM	Right middle frontal
95	GM	Right superior frontal, dorsolateral
96	WM	Right superior corona radiata
97	WM	Right corticospinal tract
98	GM	Right inferior frontal, triangular part
99	GM	Right inferior frontal, opercular part
100	GM	Left temporal pole (middle temporal)
101	GM	Left inferior occipital
102	GM	Left posterior cingulate
103	WM	Posterior limb of left internal capsule
104	GM	Left middle occipital

105	GM	Right straight
106	GM	Left middle frontal, orbital part
107	GM	Left precuneus
108	GM	Right superior frontal, medial orbital
109	GM	Left middle temporal
110	GM	Left inferior frontal, orbital part
111	GM	Right olfactory
112	GM	Left supplementary motor
113	GM	Left superior frontal, medial
114	GM	Right supplementary motor
115	GM	Left inferior frontal, triangular part
116	GM	Right posterior cingulate
117	GM	Right superior frontal, medial
118	GM	Left median cingulate and paracingulate
119	GM	Left Rolandic operculum
120	WM	Posterior limb of right internal capsule
121	GM	Left fusiform
122	GM	Left inferior frontal, opercular part
123	GM	Right anterior cingulate and paracingulate
124	GM	Right median cingulate and paracingulate

Supplementary Table 5. Staging of structural alterations according to changes in Hoehn and Yahr Stage (HYS) from < 2, via ≥ 2 and < 3, to ≥ 3 (**a**) or cognitive impairment from normal cognition (NC), via mild cognitive impairment (MCI), to dementia (**b**). *P*(X+|Y−) represents the probability of a specific brain region's structural alteration occurring earlier, whereas *P*(Y+|X−) expresses the probability of a specific brain region's structural alteration occurring later.

(**a**)

**HYS**	**Tissue**	**Brain region**	***P*(X+|Y−)**	***P*(Y+|X−)**
< 2	WM	Genu of corpus callosum	0.79	0.46
< 2	WM	Left cingulum (hippocampus)	0.73	0.48
< 2	WM	Left cingulum (cingulate gyrus)	0.71	0.43
< 2	WM	Left fornix / stria terminalis	0.70	0.43
< 2	WM	Right cingulum (hippocampus)	0.70	0.46
< 2	WM	Anterior limb of left internal capsule	0.68	0.40
< 2	WM	Retrolenticular part of left internal capsule	0.66	0.43
< 2	WM	Anterior limb of right internal capsule	0.66	0.41
< 2	WM	Left external capsule	0.65	0.42
< 2	WM	Right external capsule	0.64	0.42
< 2	GM	Right postcentral	0.64	0.38
< 2	WM	Middle cerebellar peduncle	0.61	0.42
< 2	WM	Right cingulum (cingulate gyrus)	0.61	0.42
< 2	WM	Splenium of corpus callosum	0.60	0.40
< 2	WM	Right fornix / stria terminalis	0.59	0.42
< 2	WM	Left anterior corona radiata	0.59	0.40
< 2	WM	Retrolenticular part of right internal capsule	0.58	0.42
< 2	WM	Right anterior corona radiata	0.56	0.39
< 2	WM	Left sagittal stratum	0.54	0.41
< 2	WM	Right sagittal stratum	0.53	0.40
< 2	WM	Left cerebral peduncle	0.52	0.39
< 2	GM	Left postcentral	0.52	0.36
< 2	GM	Right inferior temporal	0.51	0.39
≥ 2 and < 3	WM	Right posterior thalamic radiation	0.48	0.39
≥ 2 and < 3	WM	Left superior fronto-occipital fasciculus	0.48	0.39
≥ 2 and < 3	WM	Left superior cerebellar peduncle	0.48	0.39
≥ 2 and < 3	GM	Right pericalcarine	0.48	0.39
≥ 2 and < 3	GM	Right precentral	0.47	0.37
≥ 2 and < 3	GM	Left parahippocampal	0.46	0.38

≥ 2 and < 3	GM	Right parahippocampal	0.44	0.38
≥ 2 and < 3	GM	Right transverse temporal	0.44	0.35
≥ 2 and < 3	WM	Right superior fronto-occipital fasciculus	0.43	0.39
≥ 2 and < 3	WM	Left posterior corona radiata	0.42	0.37
≥ 2 and < 3	WM	Right superior longitudinal fasciculus	0.42	0.38
≥ 2 and < 3	WM	Left medial lemniscus	0.42	0.37
≥ 2 and < 3	WM	Pontine crossing tract	0.43	0.39
≥ 2 and < 3	GM	Right supramarginal	0.43	0.37
≥ 2 and < 3	GM	Left pericalcarine	0.43	0.37
≥ 2 and < 3	GM	Right lingual	0.43	0.38
≥ 2 and < 3	GM	Right cuneus	0.43	0.36
≥ 2 and < 3	WM	Right superior cerebellar peduncle	0.41	0.37
≥ 2 and < 3	WM	Left superior longitudinal fasciculus	0.40	0.37
≥ 2 and < 3	WM	Right inferior cerebellar peduncle	0.40	0.37
≥ 2 and < 3	WM	Body of corpus callosum	0.38	0.36
≥ 2 and < 3	WM	Left inferior cerebellar peduncle	0.39	0.37
≥ 2 and < 3	WM	Left corticospinal tract	0.40	0.39
≥ 2 and < 3	GM	Right pars orbitalis	0.40	0.36
≥ 2 and < 3	GM	Left precentral	0.39	0.34
≥ 2 and < 3	GM	Right superior temporal	0.39	0.36
≥ 2 and < 3	GM	Right superior parietal	0.37	0.35
≥ 2 and < 3	WM	Right uncinate fasciculus	0.36	0.37
≥ 2 and < 3	GM	Right lateral occipital	0.38	0.37
≥ 2 and < 3	WM	Right posterior corona radiata	0.35	0.36
≥ 2 and < 3	GM	Left lingual	0.36	0.36
≥ 2 and < 3	WM	Right cerebral peduncle	0.35	0.37
≥ 2 and < 3	GM	Left lateral orbitofrontal	0.36	0.37
≥ 2 and < 3	GM	Right middle temporal	0.37	0.38
≥ 2 and < 3	GM	Right inferior parietal	0.36	0.36
≥ 2 and < 3	WM	Fornix	0.34	0.36
≥ 2 and < 3	WM	Left superior corona radiata	0.30	0.35
≥ 2 and < 3	GM	Left transverse temporal	0.31	0.33
≥ 2 and < 3	GM	Left superior parietal	0.31	0.33
≥ 2 and < 3	WM	Left uncinate fasciculus	0.30	0.35
≥ 2 and < 3	GM	Right caudal middle frontal	0.30	0.34
≥ 2 and < 3	GM	Left caudal middle frontal	0.29	0.33

≥ 2 and < 3	GM	Left medial orbitofrontal	0.30	0.35
≥ 2 and < 3	GM	Left rostral anterior cingulate	0.31	0.36
≥ 2 and < 3	GM	Right fusiform	0.30	0.36
≥ 2 and < 3	GM	Left lateral occipital	0.29	0.33
≥ 2 and < 3	GM	Right insula	0.29	0.36
≥ 2 and < 3	GM	Right isthmus cingulate	0.29	0.35
≥ 2 and < 3	GM	Left isthmus cingulate	0.29	0.35
≥ 2 and < 3	GM	Right paracentral	0.27	0.33
≥ 2 and < 3	WM	Right medial lemniscus	0.28	0.36
≥ 2 and < 3	WM	Left tapetum	0.25	0.34
≥ 2 and < 3	GM	Right entorhinal	0.28	0.36
≥ 2 and < 3	GM	Left superior temporal	0.26	0.33
≥ 2 and < 3	GM	Left cuneus	0.26	0.35
≥ 2 and < 3	GM	Left inferior temporal	0.25	0.34
≥ 2 and < 3	GM	Right precuneus	0.24	0.34
≥ 2 and < 3	GM	Right pars opercularis	0.24	0.35
≥ 2 and < 3	GM	Left supramarginal	0.23	0.32
≥ 2 and < 3	WM	Left posterior thalamic radiation	0.22	0.34
≥ 2 and < 3	GM	Left insula	0.24	0.35
≥ 2 and < 3	GM	Left entorhinal	0.23	0.34
≥ 2 and < 3	WM	Right tapetum	0.21	0.33
≥ 2 and < 3	GM	Left middle temporal	0.22	0.34
≥ 2 and < 3	GM	Right pars triangularis	0.20	0.33
≥ 2 and < 3	GM	Left pars orbitalis	0.21	0.34
≥ 2 and < 3	GM	Right lateral orbitofrontal	0.20	0.34
≥ 2 and < 3	GM	Left rostral middle frontal	0.20	0.33
≥ 2 and < 3	WM	Right superior corona radiata	0.19	0.34
≥ 2 and < 3	GM	Left superior frontal	0.19	0.32
≥ 2 and < 3	WM	Right corticospinal tract	0.20	0.34
≥ 2 and < 3	GM	Right rostral middle frontal	0.18	0.33
≥ 2 and < 3	GM	Left inferior parietal	0.18	0.33
≥ 2 and < 3	GM	Right medial orbitofrontal	0.18	0.34
≥ 2 and < 3	WM	Posterior limb of left internal capsule	0.17	0.34
≥ 2 and < 3	GM	Left precuneus	0.17	0.33
≥ 2 and < 3	GM	Right superior frontal	0.16	0.33
≥ 2 and < 3	GM	Left pars triangularis	0.16	0.33

≥ 2 and < 3	GM	Left paracentral	0.15	0.33
≥ 2 and < 3	GM	Left posterior cingulate	0.17	0.34
≥ 2 and < 3	GM	Left caudal anterior cingulate	0.14	0.34
≥ 2 and < 3	GM	Left pars opercularis	0.12	0.33
≥ 2 and < 3	GM	Right posterior cingulate	0.11	0.33
≥ 2 and < 3	GM	Right caudal anterior cingulate	0.11	0.33
≥ 3	GM	Left fusiform	0.07	0.33
≥ 3	WM	Posterior limb of right internal capsule	0.08	0.33
≥ 3	GM	Right rostral anterior cingulate	0.12	0.34

(**b**)

**Cognitive impairment**	**Tissue**	**Brain region**	***P*(X+|Y−)**	***P*(Y+|X−)**
NC	WM	Genu of corpus callosum	0.79	0.46
NC	WM	Left cingulum (hippocampus)	0.73	0.49
NC	WM	Left cingulum (cingulate gyrus)	0.71	0.43
NC	WM	Left fornix / stria terminalis	0.70	0.43
NC	WM	Right cingulum (hippocampus)	0.70	0.46
NC	WM	Anterior limb of left internal capsule	0.68	0.40
NC	WM	Retrolenticular part of left internal capsule	0.66	0.43
NC	WM	Anterior limb of right internal capsule	0.66	0.41
NC	WM	Left external capsule	0.65	0.42
NC	WM	Right external capsule	0.64	0.42
NC	GM	Right postcentral	0.64	0.38
NC	WM	Middle cerebellar peduncle	0.61	0.42
NC	WM	Right cingulum (cingulate gyrus)	0.61	0.42
NC	WM	Splenium of corpus callosum	0.60	0.40
MCI	WM	Right fornix / stria terminalis	0.59	0.42
MCI	WM	Left anterior corona radiata	0.59	0.40
MCI	WM	Retrolenticular part of right internal capsule	0.58	0.42
MCI	WM	Right anterior corona radiata	0.56	0.39
MCI	WM	Left sagittal stratum	0.54	0.41
MCI	WM	Right sagittal stratum	0.53	0.40
MCI	WM	Left cerebral peduncle	0.52	0.39
MCI	GM	Left postcentral	0.52	0.36
MCI	GM	Right inferior temporal	0.51	0.39
MCI	WM	Right posterior thalamic radiation	0.48	0.39

MCI	WM	Left superior fronto-occipital fasciculus	0.48	0.39
MCI	WM	Left superior cerebellar peduncle	0.48	0.39
MCI	GM	Right pericalcarine	0.48	0.40
MCI	GM	Right precentral	0.47	0.37
MCI	GM	Left parahippocampal	0.46	0.38
MCI	GM	Right parahippocampal	0.44	0.38
MCI	GM	Right transverse temporal	0.43	0.35
MCI	WM	Left posterior corona radiata	0.43	0.37
MCI	WM	Right superior fronto-occipital fasciculus	0.43	0.39
MCI	WM	Right superior longitudinal fasciculus	0.42	0.38
MCI	WM	Left medial lemniscus	0.42	0.38
MCI	WM	Pontine crossing tract	0.43	0.39
MCI	GM	Right supramarginal	0.43	0.37
MCI	GM	Left pericalcarine	0.43	0.37
MCI	GM	Right lingual	0.43	0.38
MCI	GM	Right cuneus	0.43	0.36
MCI	WM	Right superior cerebellar peduncle	0.41	0.38
MCI	WM	Left superior longitudinal fasciculus	0.40	0.37
MCI	WM	Right inferior cerebellar peduncle	0.40	0.37
MCI	WM	Body of corpus callosum	0.38	0.36
MCI	WM	Left inferior cerebellar peduncle	0.39	0.37
MCI	WM	Left corticospinal tract	0.40	0.39
MCI	GM	Right pars orbitalis	0.40	0.37
MCI	GM	Left precentral	0.39	0.34
MCI	GM	Right superior temporal	0.39	0.36
MCI	GM	Right superior parietal	0.37	0.35
MCI	WM	Right uncinate fasciculus	0.36	0.37
MCI	GM	Right lateral occipital	0.38	0.37
MCI	WM	Right posterior corona radiata	0.35	0.36
MCI	GM	Left lingual	0.36	0.36
MCI	WM	Right cerebral peduncle	0.35	0.37
MCI	GM	Left lateral orbitofrontal	0.36	0.37
MCI	GM	Right middle temporal	0.37	0.38
MCI	GM	Right inferior parietal	0.36	0.36
MCI	WM	Fornix	0.33	0.36
MCI	WM	Left superior corona radiata	0.30	0.35

MCI	GM	Left transverse temporal	0.31	0.33
MCI	GM	Left superior parietal	0.31	0.33
MCI	WM	Left uncinate fasciculus	0.30	0.35
MCI	GM	Right caudal middle frontal	0.30	0.34
MCI	GM	Left caudal middle frontal	0.29	0.33
MCI	GM	Left medial orbitofrontal	0.30	0.35
MCI	GM	Left rostral anterior cingulate	0.31	0.36
MCI	GM	Right fusiform	0.30	0.36
MCI	GM	Left lateral occipital	0.29	0.33
MCI	GM	Right insula	0.29	0.36
MCI	GM	Right isthmus cingulate	0.29	0.36
MCI	GM	Left isthmus cingulate	0.29	0.35
MCI	GM	Right paracentral	0.27	0.33
MCI	WM	Right medial lemniscus	0.28	0.36
MCI	WM	Left tapetum	0.25	0.34
MCI	GM	Right entorhinal	0.28	0.36
MCI	GM	Left cuneus	0.26	0.35
MCI	GM	Left superior temporal	0.26	0.33
MCI	GM	Left inferior temporal	0.25	0.34
MCI	GM	Right precuneus	0.24	0.34
MCI	GM	Right pars opercularis	0.24	0.35
MCI	GM	Left supramarginal	0.23	0.33
MCI	WM	Left posterior thalamic radiation	0.22	0.34
MCI	GM	Left entorhinal	0.23	0.34
MCI	GM	Left insula	0.24	0.35
MCI	WM	Right tapetum	0.21	0.34
MCI	GM	Left middle temporal	0.22	0.34
MCI	GM	Right pars triangularis	0.20	0.34
MCI	GM	Left pars orbitalis	0.21	0.34
MCI	GM	Right lateral orbitofrontal	0.20	0.34
MCI	GM	Left rostral middle frontal	0.20	0.33
MCI	WM	Right superior corona radiata	0.19	0.34
MCI	GM	Left superior frontal	0.19	0.33
MCI	WM	Right corticospinal tract	0.20	0.35
MCI	GM	Right rostral middle frontal	0.18	0.34
MCI	GM	Left inferior parietal	0.18	0.33

MCI	GM	Right medial orbitofrontal	0.18	0.34
MCI	WM	Posterior limb of left internal capsule	0.17	0.34
MCI	GM	Left precuneus	0.17	0.33
MCI	GM	Right superior frontal	0.16	0.33
MCI	GM	Left pars triangularis	0.16	0.33
MCI	GM	Left paracentral	0.15	0.33
MCI	GM	Left posterior cingulate	0.17	0.34
MCI	GM	Left caudal anterior cingulate	0.14	0.34
MCI	GM	Left pars opercularis	0.12	0.33
MCI	GM	Right rostral anterior cingulate	0.12	0.34
MCI	GM	Right caudal anterior cingulate	0.11	0.33
Dementia	GM	Left fusiform	0.07	0.33
Dementia	WM	Posterior limb of right internal capsule	0.08	0.33
Dementia	GM	Right posterior cingulate	0.11	0.33

GM, grey matter; and WM, white matter.

Supplementary Table 6. Lists of 62 cortical grey matter (GM) regions (**a**) and 48 white matter (WM) regions (**b**) for which cortical thinning and WM disintegrity, respectively, were assessed.

(**a**)

No	Cortical GM region
1	Left caudal middle frontal
2	Left entorhinal
3	Left postcentral
4	Left pars triangularis
5	Left supramarginal
6	Left insula
7	Left lateral orbitofrontal
8	Left pars orbitalis
9	Left middle temporal
10	Left pericalcarine
11	Left parahippocampal
12	Left paracentral
13	Left medial orbitofrontal
14	Left cuneus
15	Left inferior temporal
16	Left rostral middle frontal
17	Left rostral anterior cingulate
18	Left isthmus cingulate
19	Left lateral occipital
20	Left lingual
21	Left superior parietal
22	Left pars opercularis
23	Left fusiform
24	Left caudal anterior cingulate
25	Left superior frontal
26	Left precuneus
27	Left transverse temporal
28	Left precentral
29	Left inferior parietal
30	Left posterior cingulate
31	Left superior temporal

32	Right caudal middle frontal
33	Right entorhinal
34	Right postcentral
35	Right pars triangularis
36	Right supramarginal
37	Right insula
38	Right lateral orbitofrontal
39	Right pars orbitalis
40	Right middle temporal
41	Right pericalcarine
42	Right parahippocampal
43	Right paracentral
44	Right medial orbitofrontal
45	Right cuneus
46	Right inferior temporal
47	Right rostral middle frontal
48	Right rostral anterior cingulate
49	Right isthmus cingulate
50	Right lateral occipital
51	Right lingual
52	Right superior parietal
53	Right pars opercularis
54	Right fusiform
55	Right caudal anterior cingulate
56	Right superior frontal
57	Right precuneus
58	Right transverse temporal
59	Right precentral
60	Right inferior parietal
61	Right posterior cingulate
62	Right superior temporal

(b)

**No**	**WM region**
1	Middle cerebellar peduncle
2	Pontine crossing tract
3	Genu of corpus callosum

4	Body of corpus callosum
5	Splenium of corpus callosum
6	Fornix
7	Left corticospinal tract
8	Right corticospinal tract
9	Left medial lemniscus
10	Right medial lemniscus
11	Left inferior cerebellar peduncle
12	Right inferior cerebellar peduncle
13	Left superior cerebellar peduncle
14	Right superior cerebellar peduncle
15	Left cerebral peduncle
16	Right cerebral peduncle
17	Anterior limb of left internal capsule
18	Anterior limb of right internal capsule
19	Posterior limb of left internal capsule
20	Posterior limb of right internal capsule
21	Retrolenticular part of left internal capsule
22	Retrolenticular part of right internal capsule
23	Left anterior corona radiata
24	Right anterior corona radiata
25	Left superior corona radiata
26	Right superior corona radiata
27	Left posterior corona radiata
28	Right posterior corona radiata
29	Left posterior thalamic radiation
30	Right posterior thalamic radiation
31	Left sagittal stratum
32	Right sagittal stratum
33	Left external capsule
34	Right external capsule
35	Left cingulum (cingulate gyrus)
36	Right cingulum (cingulate gyrus)
37	Left cingulum (hippocampus)
38	Right cingulum (hippocampus)
39	Left fornix / stria terminalis

40	Right fornix / stria terminalis
41	Left superior longitudinal fasciculus
42	Right superior longitudinal fasciculus
43	Left superior fronto-occipital fasciculus
44	Right superior fronto-occipital fasciculus
45	Left uncinate fasciculus
46	Right uncinate fasciculus
47	Left tapetum
48	Right tapetum

		Y	
		+	-
X	+	*a*	*b*
	-	*c*	*d*

$$P(X+|Y+) = \frac{a}{a+c} \qquad P(Y+|X+) = \frac{a}{a+b}$$

$$P(X+|Y-) = \frac{b}{b+d} \qquad P(Y+|X-) = \frac{c}{c+d}$$

$$P(X-|Y+) = \frac{c}{a+c} \qquad P(Y-|X+) = \frac{b}{a+b}$$

$$P(X-|Y-) = \frac{d}{b+d} \qquad P(Y-|X-) = \frac{d}{c+d}$$

Supplementary Figure 1. Conditional probability using a two-way table. When the presence (+) or absence (-) of a structural alteration in two brain regions, X and Y, is considered, *a*, *b*, *c*, and *d* represent the total amount of observations across patients for different cases. The observations of structural alterations can be formulated as the probabilistic relationship of the events between the brain regions, that is, the conditional probability of an event, given another event. Among conditional probabilities that express possible probabilistic relationships between the brain regions, as displayed below the two-way table, conditional probabilities of discordant events, *P*(X+|Y-) and *P*(Y+|X-) (shaded ones), are specifically informative about the ordering of structural alterations between the brain regions, since they could provide the evidence of a structural alteration tending to occur earlier in one brain region than in another brain region. Conditional probability can be computed by the relative frequency that an event occurred, in relation to the times that another event occurred: *P*(X+|Y-) by the fraction of X+ observations among Y- observations, namely, $P(X+|Y-) = P(X+ \cap Y-)/P(Y-) = b/(b+d)$; and similarly, *P*(Y+|X-) by the fraction of Y+ observations among X- observations, namely, $P(Y+|X-) = P(Y+ \cap X-)/P(X-) = c/(c+d)$.

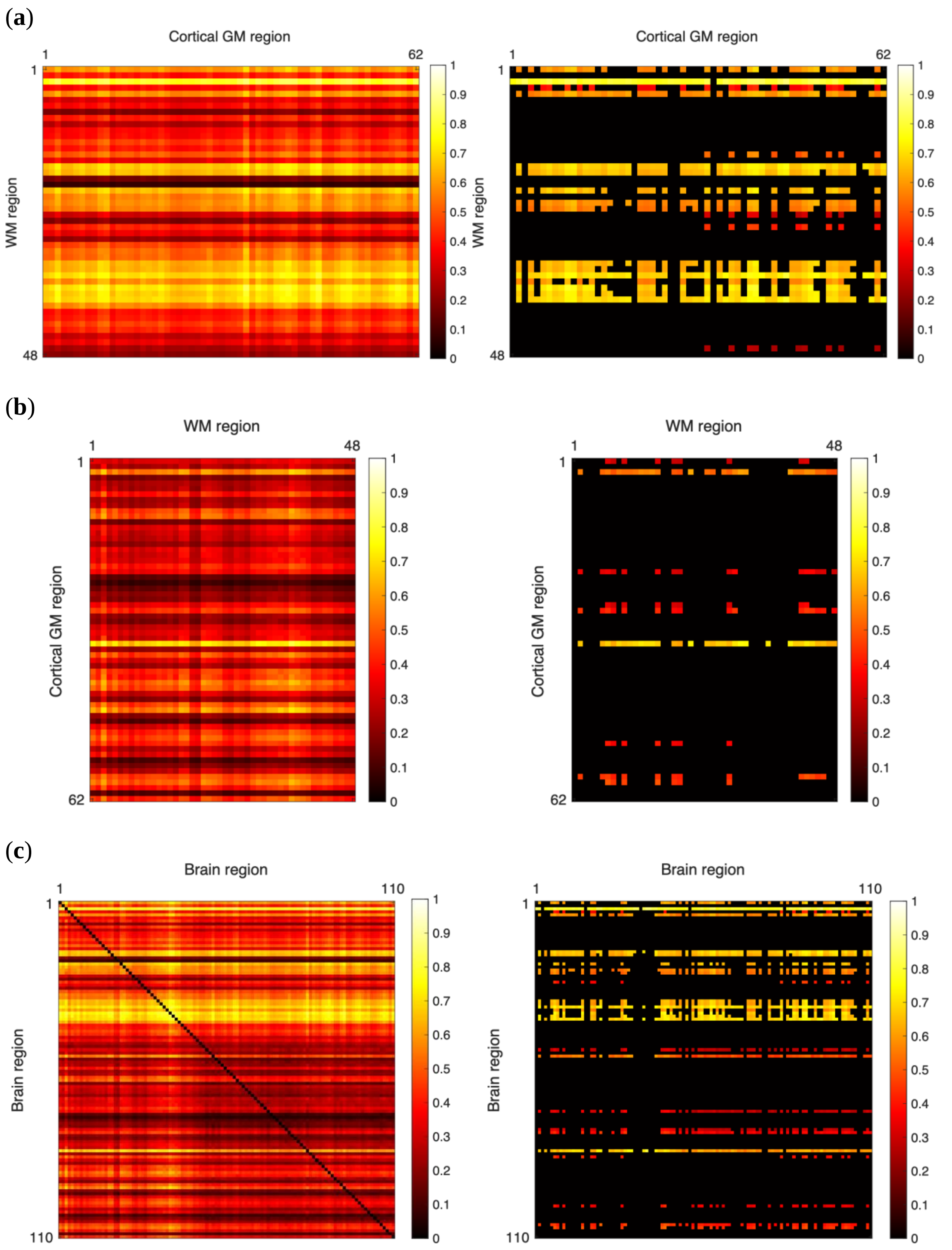

Supplementary Figure 2. Matrices of conditional probabilities for white matter (WM) disintegrity preceding cortical thinning (48 WM regions × 62 cortical grey matter (GM) regions) (**a**), cortical thinning preceding WM disintegrity (62 cortical GM regions × 48 WM regions) (**b**), and the precedence of structural alterations (110 brain regions × 110 brain

regions) (**c**). In **a** and **b**, 62 cortical GM regions and 48 WM regions are listed as numbered in Supplementary Table 6, and in **c**, 110 brain regions are listed with 48 WM regions followed by 62 cortical GM regions. Conditional probabilities were mapped for all regional pairs in the left panels, whereas they were mapped only for regional pairs of statistical significance in the right panels. Of all regional pair combinations, 24% (702 of 2976) were significant in **a**, 5% (142 of 2976) were significant in **b**, and 14% (1643 of 11990) were significant in **c**.

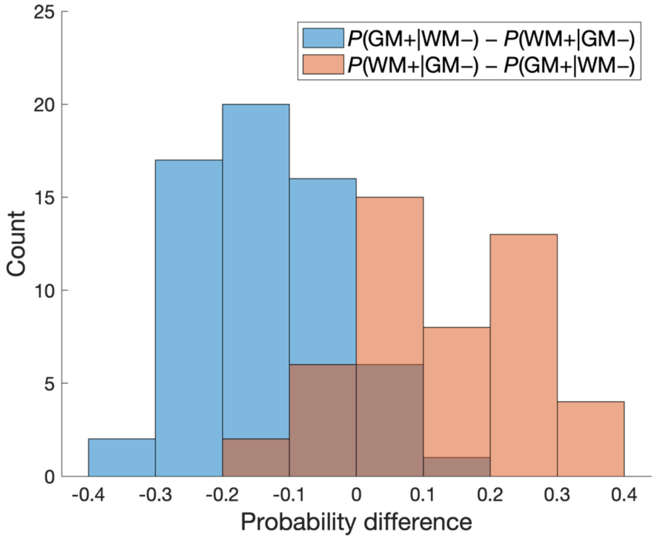

Supplementary Figure 3. Distribution of values of the difference between *P*(GM+|WM−) and *P*(WM+|GM−) in estimating the ordering of structural alterations between cortical grey matter regions and whiter matter regions.

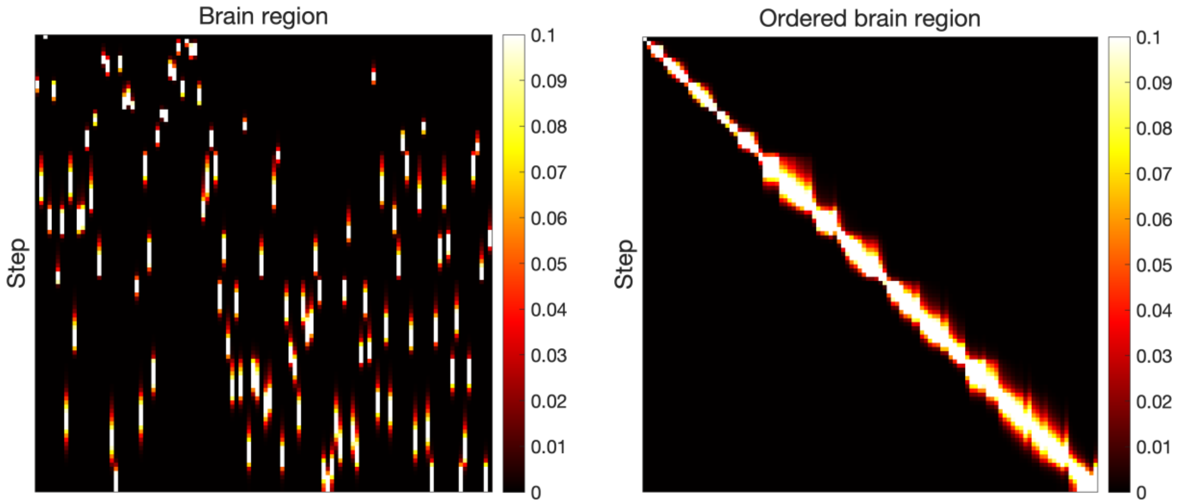

Supplementary Figure 4. Matrices of the relative frequency of each brain region's structural alteration occurring at the respective step of simulated sequences in estimating the ordering of structural alterations across 110 brain regions. Rows from top to bottom indicate steps of simulated sequences and columns from left to right represent brain regions in the left panel and those ordered according to the selected sequence of structural alterations in the right panel. The distribution of frequencies implies variances in brain regions structural alterations of which would occur at a specific step as well as variances in steps at which a specific brain region's structural alteration would occur.

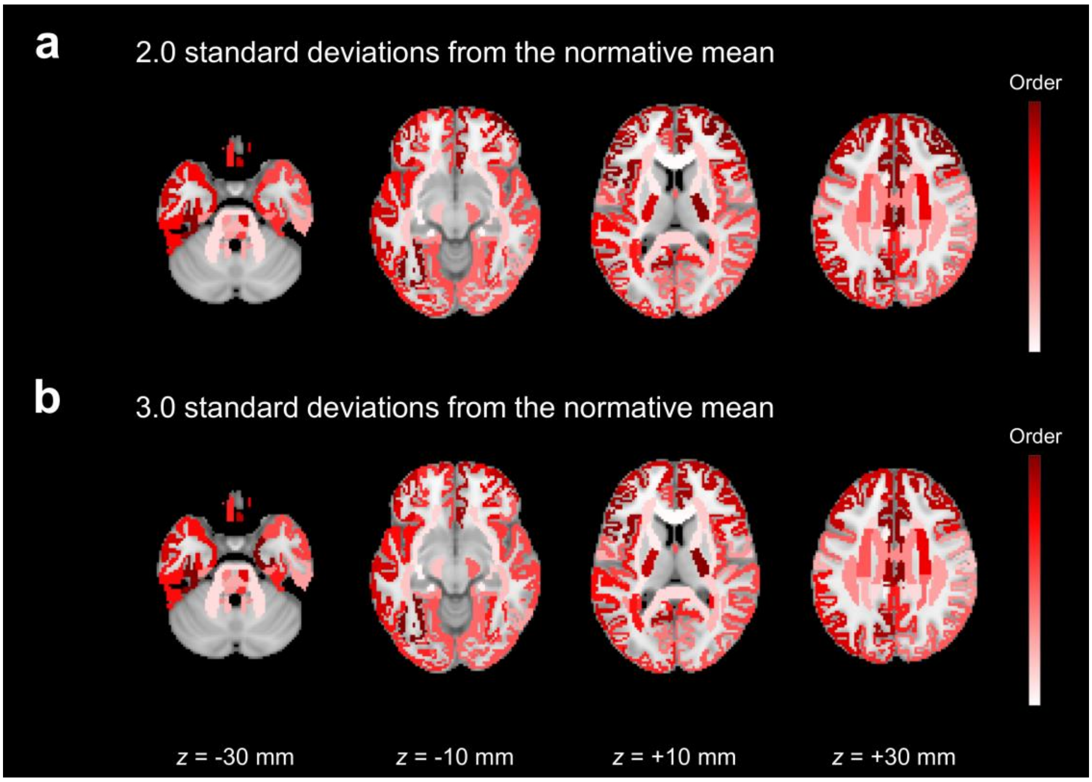

Supplementary Figure 5. Ordering of structural alterations across 110 brain regions in the case of applying the cut-off of 2.0 (**a**) and 3.0 (**b**) standard deviations, instead of the default choice of 2.5 standard deviations, from the mean of normative controls' values in determining structural alterations in the brain regions. The white to maroon color scheme corresponds to the earlier to later occurrence of structural alterations.

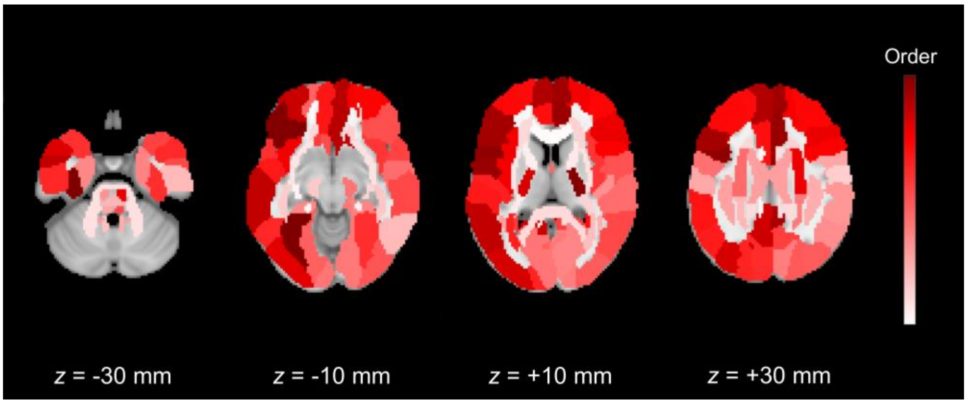

Supplementary Figure 6. Ordering of structural alterations across 124 brain regions in the case of parcellating the brain into 76 cortical grey matter regions, instead of the default choice of 62 ones, and 48 white matter regions. The white to maroon color scheme corresponds to the earlier to later occurrence of structural alterations.

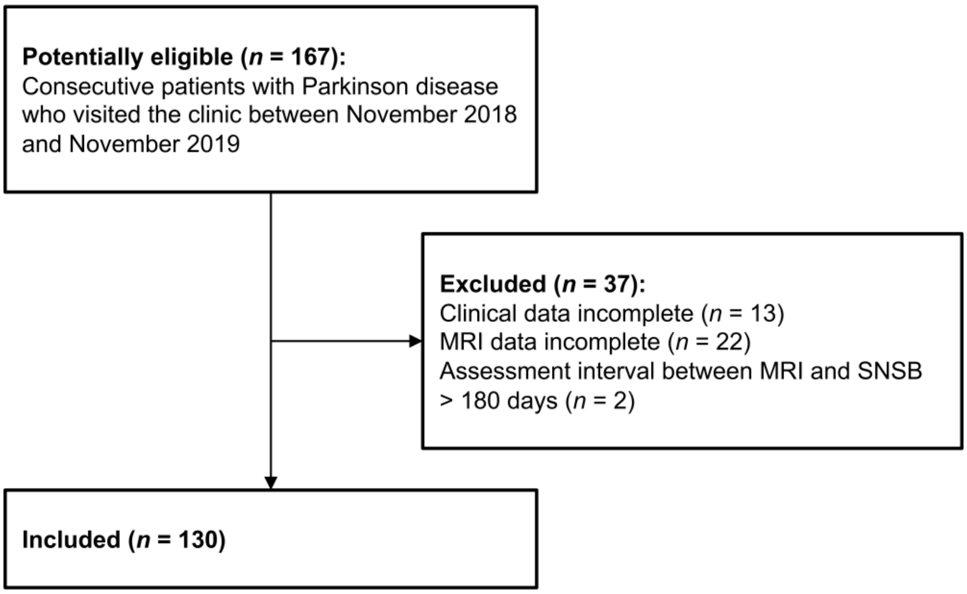

Supplementary Figure 7. Flowchart of patient inclusion and exclusion in the study. SNSB, Seoul Neuropsychological Screening Battery.
